# Wet Chemistry Route to Li_3_InCl_6_: Microstructural Control Render High Ionic Conductivity and Enhanced All‐Solid‐State Battery Performance

**DOI:** 10.1002/advs.202403208

**Published:** 2024-07-08

**Authors:** Jacob Otabil Bonsu, Abhirup Bhadra, Dipan Kundu

**Affiliations:** ^1^ School of Chemical Engineering UNSW Sydney Kensington NSW 2052 Australia; ^2^ School of Mechanical and Manufacturing Engineering UNSW Sydney Kensington NSW 2052 Australia

**Keywords:** all‐solid‐state lithium battery, aprotic solvent‐mediated scalable synthesis, halide solid electrolyte, high ionic conductivity, microstructural characteristics

## Abstract

Thanks to superionic conductivity and compatibility with >4 V cathodes, halide solid electrolytes (SEs) have elicited tremendous interest for application in all‐solid‐state lithium batteries (ASSLBs). Many compositions based on groups 3, 13, and divalent metals, and substituted stoichiometries have been explored, some displaying requisite properties, but the Li^+^ conductivity still falls short of theoretical predictions and appealing sulfide‐type SEs. While controlling microstructural characteristics, namely grain boundary effects and microstrain, can boost ionic conductivity, they have rarely been considered. Moving away from the standard solid‐state route, here a scalable and facile wet chemical approach for obtaining highly conductive (>2 mS cm^−1^) Li_3_InCl_6_ is presented, and it is shown that aprotic solvents can reduce grain boundaries and microstrain, leading to very high ionic conductivity of over 4 mS cm^−1^ (at 22 °C). Minimized grain boundary area renders improved moisture stability and enhances solid–solid interfacial contact, leading to excellent LiNi_0.6_Mn_0.2_Co_0.2_O_2_‐based full‐cell performance, exemplified by stable room temperature (22 °C) cycling at a 0.2 C rate with 155 mAh g^−1^ capacity and 85% retention after 1000 cycles at 60 °C with a high 99.75% Coulombic efficiency. The findings showcase the viability of the aprotic solvent‐mediated route for producing high‐quality Li_3_InCl_6_ for all‐solid‐state batteries.

## Graphical abstract

An easily scalable wet chemical method is presented to obtain highly functional Li_3_InCl_6_ solid electrolytes. Aprotic solvents yield favorable microstructural characteristics, with tetrahydrofuran rendering a record high ionic conductivity of >4 mS cm^−1^ at 22 °C. The optimal microstructure leads to the least resistive and stable interfaces in the solid‐state battery cathode, enabling excellent performance at room temperature and 60 °C.

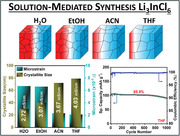

## 1 Introduction

Improved safety and the potential for high energy density and extended lifespan have inspired tremendous research and commercial interests in ASSLBs.^[^ ^1^, ^2^ ^]^ The difference from conventional Li‐ion batteries stems from the use of inorganic SEs with superionic single Li‐ion conductivity, high thermal stability, and excellent mechanical properties.^[^ ^3^ ^]^ Hence, the efficacy of solid‐state batteries predominantly hinges upon the optimal functioning of SEs and their ability to facilitate facile ion conduction, seamless ionic contact, and electrochemical and mechanical stability across interfaces.^[^ ^4^ ^]^

During the past two decades, significant advancements have been made in designing SEs with different chemistries, namely borohydride,^[^ ^5^ ^]^ phosphate, oxide,^[^ ^6^, ^7^, ^8^ ^]^ sulfide,^[^ ^3^, ^9^ ^]^ and halide.^[^ ^10^, ^11^, ^12^ ^]^ The last type – with their general formula of Li_z_MX_2z_ (where M: Sc, Y, In, Zr, Hf, etc., and X: F/Cl/Br) – is promising owing to high Li^+^ conductivity (>1 mS cm^−1^),^[^ ^13^ ^]^ oxidative stability and high‐voltage cathode compatibility,^[^ ^14^ ^]^ good air stability,^[^ ^12^, ^15^, ^16^, ^17^, ^18^ ^]^ and mechanical properties favorable for processing of electrolyte membranes by cold pressing.^[^ ^14^, ^18^ ^]^ Electrochemical stability at higher voltages imparts significant interfacial stability with 4 V‐class cathode active materials, eliminating the need for cathode particle coating.^[^ ^19^ ^]^ Nevertheless, the ionic conductivities of halide SEs still fall short of theoretical predictions and lag behind some of the promising sulfide SEs. Microstrain and grain boundary control can enhance ionic conductivity and improve ionic contact at numerous solid–solid interfaces, leading to improved solid‐state battery performance, but remain a rarely explored strategy.^[^ ^20^ ^]^

The wet chemical method – a scalable and potentially inexpensive route to SE synthesis – can allow significant control over the size and quality of the crystallites and, hence, the extent of grain boundaries and localized microstrain.^[^ ^21^ ^]^ Yet, until Sun et al. synthesized Li_3_InCl_6_ from aqueous media,^[^ ^13^ ^]^ almost all halide SEs were synthesized via the solid‐state reaction routes, namely mechanochemical milling,^[^ ^14^ ^]^ mechanical milling with post‐annealing^[^ ^22^ ^]^ and solid‐state sintering,^[^ ^23^, ^24^ ^]^ which are energy intensive, challenging to scale up,^[^ ^1^ ^]^ and results in Li_3_InCl_6_ with smaller particle size and low ionic conductivity (Table S1, Supporting Information). This is partly because solvent‐mediated synthesis can lead to the hydrolysis of precursors or complex formation, resulting in impurities. For instance, Sun et al. tried to synthesize Li_3_YCl_6_ via the wet‐chemistry approach with various solvents that can dissolve YCl_3_ and LiCl. However, the X‐ray diffraction (XRD) revealed the presence of LiCl and yttrium oxychloride (YOCl) as impurities.^[^ ^25^ ^]^ Tu et al. synthesized Li_3_InCl_6_ from ethanol but struggled to attain optimum ionic conductivity with an enormous grain boundary effect, significantly affecting the cell performance.^[^ ^26^ ^]^

Herein, we present a facile and scalable wet chemical approach for synthesizing highly conducting (>2 mS cm^−1^) Li_3_InCl_6_ SE from both protic (water/H_2_O and ethanol/EtOH) and aprotic (acetonitrile/ACN and tetrahydrofuran/THF) solvents. However, it is the aprotic solvent‐mediated synthesis that leads to the formation of Li_3_InCl_6_ with large crystallite size and agglomerated morphology and, thus, the least grain boundary contributions and minimum microstrain, resulting in a record high ionic conductivity (4 mS cm^−1^) ever reported experimentally for the halide type SEs. Owing to the reduced grain boundary, the THF‐derived Li_3_InCl_6_ also displays the least degradation on exposure to humidity. Thanks to the desired SE characteristics and excellent interfacial contact and stability, the LiNi_0.6_Mn_0.2_Co_0.2_O_2_ (NMC622) based ASSLBs assembled with THF‐derived Li_3_InCl_6_ demonstrate excellent electrochemical performance – highlighted by 100% retention of 155 mAh g^−1^ capacity after 100 cycles at a 0.2 C rate and a remarkable 85% capacity retention over 1000 cycles at a 2 C rate at 60 °C.

## 2 Results and Discussion

**Figure 1:**
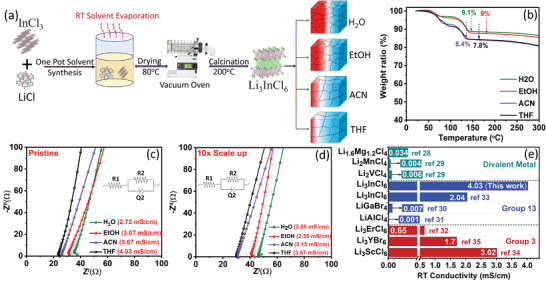
a) Schematic representation of the wet chemical synthesis of Li_3_InCl_6_ SEs. b) Thermogravimetric analysis (TGA) of the intermediates obtained after the 80 °C drying step. Nyquist impedance plots for the c) wet chemical route derived Li_3_InCl_6_ SEs and d) the 10x scaled‐up THF‐derived samples. e) Comparison of the ionic conductivity of the Li_3_InCl_6_ SE synthesized using THF with several reported Li‐ion conducting halide SEs measured at room temperature (22 °C) on cold‐pressed pellets.

Synthesis of Li_3_InCl_6_ solid electrolyte was successfully achieved via the wet chemical method using four different solvents: tetrahydrofuran (THF), acetonitrile (ACN), ethanol (EtOH), and water (H_2_O). These protic (H_2_O and ethanol) and aprotic (THF and ACN) solvents with high dielectric constants were chosen due to the ease of precursor dissolution.^[^ ^27^ ^]^ A schematic illustration of the synthesis method is presented in **Figure** **1**a. LiCl and InCl_3_ were taken in a 3:1 stoichiometric ratio and stirred until their dissolution. The solvent was then evaporated at room temperature (22 °C) without applying any heat until the solutions became well‐saturated for evaporative crystallization to commence. Several factors, including atmospheric pressure, airflow above the solution, solubility of solutes, presence of impurities, temperature, and solvent properties, tend to affect the rate of evaporative crystallization. Among these, temperature regulation and the choice of solvent, which are critical for controlling the quality and properties of the obtained crystallites^[^ ^28^ ^]^ were considered here. Crystallites obtained by evaporation were vacuum‐dried at 80 °C for 45 min and subsequently calcined at 200 °C for 2 h to obtain pure Li_3_InCl_6_ SEs. The calcination temperature for the dried pulverized intermediate was decided based on thermogravimetric analysis as shown in Figure 1b, which shows a weight loss of 7.8, 8.4, 9, and 9.1% for THF, ACN, EtOH, and H_2_O mediated synthesis, respectively, prior to stabilization ≈150–200 °C. A greater weight loss for the protic solvent‐derived intermediates suggests the formation of intermediate complexes with higher solvent stoichiometries. Nevertheless, the wet chemical approach is highly scalable (Figure S1, Supporting Information) and can produce 3–4 g (ten times that of the typical lab‐scale synthesis) or more Li_3_InCl_6_ per batch. As discussed below, this scalability is feasible with all four solvents, producing solid electrolytes with a high purity.

The ionic conductivities of the as‐synthesized Li_3_InCl_6_ SEs were measured on cold‐pressed pellets using electrochemical impedance spectroscopy (EIS). The corresponding Nyquist plots for the small‐scale and 10x scaled‐up (synthesis) samples are shown in Figure 1c,d, respectively. As expected for the blocking electrode configuration, all samples exhibit a characteristic sloping line in their impedance spectra. The corresponding impedance values were derived from the equivalent circuit fitting, as shown in the inset of Figure 1c,d. This equivalent circuit model was consistently applied for the EIS analysis of SE samples unless otherwise specified. High ionic conductivities of 4.03, 3.67, 3.07, and 2.72 mS cm^−1^ for the small‐scale and 3.67, 3.13, 2.35, and 2.08 mS cm^−1^ for 10x scaled‐up Li_3_InCl_6_ SEs, synthesized from THF, ACN, EtOH, and H_2_O, respectively, are obtained from the equivalent circuit fitting which typically matched with the Z′ (real Z) axis intercept of the impedance data. While the observed high ionic conductivities can be attributed to the high bulk conductivity and low grain boundary resistance, the difference between the SEs synthesized from different solvents was thought to stem from differences in grain boundary characteristics (crystallite size). The selection of the solvent in the solution‐mediated process can influence the rate at which crystallization occurs. Solvents with lower boiling points undergo fast evaporation at room temperature and facilitate accelerated crystallization, which is conducive to the growth of larger crystallites and, hence, lower grain boundary area. The same can be induced by increasing the evaporation temperature, but high temperatures may also give rise to impurities or unfavorable crystal structures. Between ACN and EtOH, even though the former has a slightly higher boiling point, the high volatility of ACN (higher vapor pressure at room temperature than EtOH)^[^ ^29^ ^]^ at around room temperature leads to faster crystallization of the Li_3_InCl_6_ SE, yielding larger crystallite size (see below) and hence lower grain boundary effect. Notably, to the best of our knowledge, the room ionic conductivity of 4.03 mS cm^−1^ achieved for THF‐derived Li_3_InCl_6_ SE is the highest conductivity ever reported for halide SEs (Figure 1e).^[^ ^30^, ^31^, ^32^, ^33^, ^34^, ^35^ ^]^

**Figure 2:**
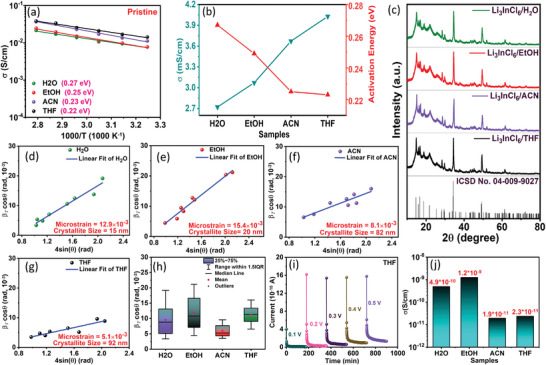
a) Arrhenius fitting of the temperature‐dependent conductivity data for pristine Li_3_InCl_6_ SEs. b) The room temperature ionic conductivity and the ion migration barrier (activation energy) of the SEs as a function of the solvent used for the solution‐mediated synthesis. c) Slow‐scanned powder XRD patterns of the as‐synthesized SEs. The Williamson–Hall plot of the SEs derived using d) H_2_O, e) EtOH, f) ACN, and g) THF. h) Box plot of the full width at half maximum (FWHM) values of the XRD peaks for the SEs samples. i) DC polarization curves of Li_3_InCl_6_ SEs, synthesized using THF, obtained by applying different voltage biases in a symmetric cell configuration. j) Comparison of the electronic conductivity of the SEs derived using different solvents.

As evident from the Arrhenius plots in **Figure** **2**a, Li_3_InCl_6_, synthesized using aprotic THF and ACN, exhibits slightly smaller activation energies of 0.22 and 0.23 eV, respectively, compared to that for EtOH (0.25 eV), and H_2_O (0.27 eV) derived samples. This is consistent with the observed ionic conductivity trend (Figure 2b). Nevertheless, the temperature‐dependent conductivity data for all samples demonstrate a linear trend, confirming that no phase change or deterioration occurs in the measured temperature range, which is expected. The Nyquist plots and ionic conductivities obtained at various temperatures ranging from 25 to 85 °C are presented in Figure S2 and Table S2 (Supporting Information). Additionally, Figure S3 (Supporting Information) showcases comparative Nyquist profiles at different temperatures for better visualization. As with the small‐scale synthesis, for the scaled‐up synthesis, SEs prepared from THF and ACN exhibit lower activation energies than those prepared from H_2_O and EtOH, but the disparity in activation energy values between protic and aprotic solvents is considerably greater (Figure S4, Supporting Information). Figures S5 and S6 and Table S3 (Supporting Information) show the corresponding Nyquist plots and ionic conductivities for the 10x samples as a function of temperature, which are akin to the observation for the small‐scale samples.

The powder X‐ray diffraction (XRD) analysis was carried out to assess the phase purity of the as‐synthesized materials, which is presented in Figure 2c and Figure S7 (Supporting Information) (10x samples). The diffraction data for all samples correspond well to the monoclinic C2/m (ICSD No. 04‐009‐9027) phase of Li_3_InCl_6_. Notably, there are no precursor impurities, i.e., that of LiCl or InCl_3_, nor any impure metal oxychloride phases, indicating that the synthesis results in a pure phase. X‐ray photoelectron spectroscopy (XPS) confirmed the absence of any surface impurities that could still impact the observed ionic conductivities (Figure S8, Supporting Information). The Williamson–Hall method (see the Supporting Information) was employed to estimate the crystallite sizes and induced microstrain in samples prepared using different solvents, and the corresponding data are shown in Figure 2d–g and Figure S9 (Supporting Information) (10x samples). The crystallite size and microstrain are interconnected parameters that delineate the SE's microstructural characteristics and profoundly influence the ionic conductivity. The concept of crystallite size pertains to the dimensions of discrete crystalline domains or grains present within a polycrystalline material.^[^ ^36^ ^]^ In polycrystalline SEs, grain boundaries are known to impede the movement of ions considerably. Larger crystallite sizes result in fewer grain boundaries and permit less inhibited movement of ions through the bulk of the larger crystallite, thus eventually reducing the overall impedance to ion migration. Microstrain, which refers to imperfections or local distortions inside a material's crystal lattice, has a similar effect.^[^ ^37^ ^]^ Higher microstrain levels can induce localized variations in the interatomic distances within the crystal lattice, and these regions can impede the movement of ions and impact the ionic conductivity.

As evident from Figure 2d–g, ACN and THF‐derived SEs display a much larger crystallite size of 82 and 92 nm compared to H_2_O (15 nm) and EtOH (20 nm) derived ones. The aprotic solvents also lead to a lower microstrain (ACN: 8.1 × 10^−3^, THF: 5.1 × 10^−3^) than the protic ones (H_2_O: 12.9 × 10^−3^, EtOH: 15.4 × 10^−3^). These observations are fully consistent with the observed ionic conductivities, and the high ionic conductivity of THF‐derived Li_3_InCl_6_ SE can be ascribed to the combined influence of large crystallite size and low microstrain. The same trend is evident in the 10x scaled‐up samples (Figure S9, Supporting Information), but the slightly lower ionic conductivity observed here for THF and ACN can also be explained in terms of the reduced crystallite size. The box plot in Figure 2h and Figure S10 (Supporting Information) displays how the FWHM of the diffraction peaks, used for the microstrain and crystallite size analysis, is distributed for the individual solvents. From the plots, it can be seen that all the FWHM data sets used for the analysis (microstrain and crystallite size) exhibit values within their respective interquartile (or IQR) ranges without any outliers. This finding supports the accuracy of the approach employed for the above analysis. The electronic conductivity of the SEs was determined by the direct current (DC) polarization measurement (Figure 2i; Figure S11, Supporting Information), and the estimated electronic conductivity values for the H_2_O, EtOH, ACN, and THF derived SEs are 4.9 × 10^−10^, 1.2 × 10^−9^, 1.9 × 10^−11^ and 2.3× 10^−11^ S cm^−1^ (Figure 2j; Figure S12, Supporting Information) respectively. These values are, as expected, low and confirm the absence of any electrically conductive impurities and, thus, the feasibility of the synthetic approach. The high ionic conductivity and low electronic conductivity, as observed here, are essential qualities of promising solid electrolytes for practical implementation in solid‐state batteries.^[^ ^38^ ^]^

**Figure 3:**
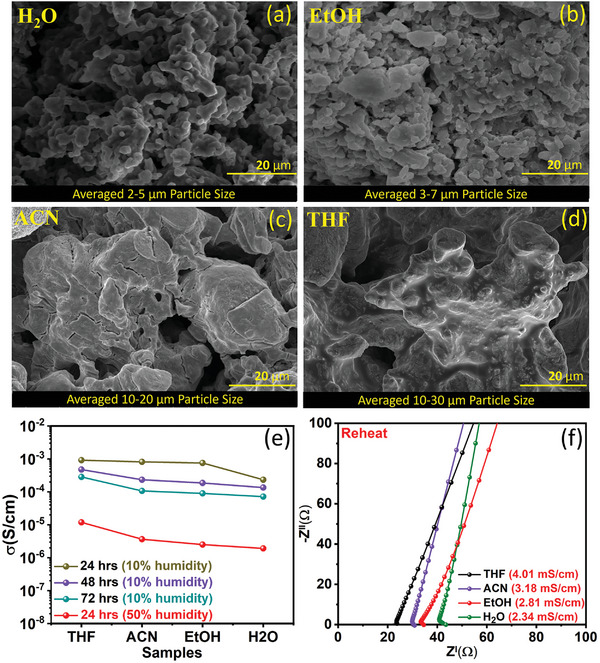
Representative SEM images of the as‐synthesized Li_3_InCl_6_ SEs derived using a) H_2_O, b) EtOH, c) ACN, and d) THF. e) Ionic conductivity evolution of the SE samples exposed in 10% and 50% humidity for different durations. f) Nyquist impedance plots for the reheated SE samples that were exposed to humidity.

The scanning electron microscopy (SEM) images in Figure 3a–d depicts the typical morphology of the as‐synthesized SEs. The particle sizes are estimated to be in the range of 2–5, 3–7, 10–20, and 10–30 µm for H_2_O, EtOH, ACN, and THF‐derived SEs, respectively. Interestingly, this observation aligns with the variation in the crystallite size. Most notably, contrary to the particulate morphology observed for H_2_O and EtOH, THF and ACN render a highly agglomerated morphology that is likely to lead to lesser grain boundaries and grain boundary resistance. A similar particle size and morphology variation trend is also observed for the 10x scaled‐up synthesis, as shown in Figure S13 (Supporting Information).

The ionic conductivity and structural evolution of the Li_3_InCl_6_ SEs were further investigated after being subjected to 10% and 50% humidity for different durations. An ionic conductivity of ≈10^−3^ S cm^−1^ and over 10^−4^ S cm^−1^ is still retained after 24 and 48/72 h exposure to an environment with 10% humidity (Figure 3e). The precise conductivity values are presented in Table S4 (Supporting Information), and the corresponding Nyquist plots are shown in Figure S14 (Supporting Information). As evident from Figure S15 (Supporting Information), the primary XRD peaks for the Li_3_InCl_6_ are still visible even after the 24 h exposure. However, the SEs synthesized from EtOH and H_2_O exhibit a minor presence of the hydrolyzed Li_3_InCl_6_, most likely due to the smaller particle and crystallite size, which render them more susceptible to moisture. When the humidity level is increased to 50%, the conductivities reduce significantly after 24 h, as shown in Figure 3e and Table S5 (Supporting Information). The Nyquist plots shown in Figure S16 (Supporting Information) clearly show the large rise in grain boundary resistance as the interfaces, boundary layers, and bulk get soaked with moisture, eventually degrading the conductive monoclinic phase of Li_3_InCl_6_. Consistent with the large crystallite size and large agglomerated morphology of the THF‐derived sample, the impedance rise is considerably smaller than for the other samples. Nonetheless, heating the degraded samples at 200 °C under vacuum for 1 h can recover the original Li_3_InCl_6_ monoclinic phase (Figure S15a–d, Supporting Information) with almost the same ionic conductivity values as the as‐synthesized SEs (Figure 3f). This reversible degradation‐revival places Li_3_InCl_6_ at a significant advantage compared to sulfide and garnet‐type solid electrolytes in terms of ease of handling and implementation in future solid‐state batteries.

**Figure 4:**
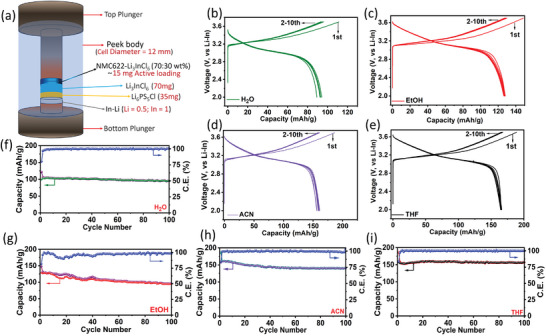
a) Schematic illustration of ASSLB cell setup. The electrochemical performance of Li‐In/Li_6_PS_5_Cl//Li_3_InCl_6_/NMC622 all‐solid‐state cells was evaluated at 22 ^o^C. The first ten charge–discharge curves at 0.2 C for cells with Li_3_InCl_6_ synthesized from ‐ b) H_2_O, c) EtOH, d) ACN, and e) THF. Cycling stability of the cells at 0.2 C with Li_3_InCl_6_ SE synthesized from f) H_2_O, g) EtOH, h) ACN, and i) THF.

In light of the highly desired characteristics exhibited by the wet chemical synthesized Li_3_InCl_6_ solid electrolytes, they were paired against LiNi_0.6_Mn_0.2_Co_0.2_ (or NMC622) in an ASSLB to assess their performance. **Figure** **4**a schematically illustrates the battery cell setup, in which a thin layer of Li_6_PS_5_Cl was incorporated between the Li_3_InCl_6_ SE layer and the lithium–indium (Li_x_In, x≤0.5) alloy (anode) to facilitate a stable anode interface.^[^ ^25^ ^]^ Details regarding the assembly of the cells are provided in the experimental section (Supporting Information). For brevity, here, we will refer to Li_3_InCl_6_ SEs by the name of the respective solvents used for their synthesis. Figures 4b–e show the first ten charge–discharge profiles at room temperature (22 °C) at 0.2 C. THF and ACN cells display the highest first‐cycle discharge capacity of ≈155 mAh g^−1^. In contrast, the EtOH cell delivers a slightly lower 129 mAh g^−1^, and the H_2_O cell delivers an underwhelming 99 mAh g^−1^. Otherwise, all cells show nearly identical and stable polarization profiles. Longer‐term cycling data, as shown in Figure 4f–i, reveal stable behavior for all cells, with H_2_O, EtOH, ACN, and THF cells exhibiting a reversible capacity of 95, 95, 140, and 156 mAhg^−1^, equivalent to a capacity retention of 96%, 74%, 90%, and 100% (Figure S17, Supporting Information) respectively, after 100 cycles. The low specific capacity for the H_2_O cell and the inferior capacity retention for the EtOH cell presumably stem from combined deleterious effects of the large cathode – SE particles interfacial area in the cathode composite and high SE microstrain, respectively. This hypothesis is supported by the EIS analyses of the cathode composites, as presented in Figures S18a,b and Table S6 (Supporting Information). While the aprotic solvent‐derived SEs lead to a rather small interfacial resistance (or cathode internal resistance; ≤15 Ω), the water‐derived SE displays the highest resistance (≈65 Ω) followed by the ethanol‐derived SE (≈38 Ω). The high internal resistance for the water‐derived SE can be correlated with the smallest crystallite size of the SE material, which manifests as the largest cathode – SE interfacial area in the cathode. Interfacial degradation – unavoidable even for oxidatively stable Li_3_InCl_6_ – and the resistive degradation product will add to the internal resistance.^[^ ^39^ ^]^ The EIS data also depicts a noticeable rise in the cathode internal resistance upon resting for the protic solvent‐derived SEs, especially for EtOH. A high microstrain, which reflects the high degree of defects in the crystallites for the EtOH‐derived Li_3_InCl_6_, leads to enhanced cathode interfacial degradation. This contributes to the observed cathode internal resistance buildup initially and explains the observed specific capacity decay for the EtOH cell upon cycling.

The capacity retention (≈100%) is particularly remarkable for THF, which, along with a very high average Coulombic efficiency of 99.98% (Figure S19, Supporting Information), confirms the quality of the THF‐derived Li_3_InCl_6_ SE. The stability for THF and ACN cells is further evident from the differential capacity (dQ/dV) plots shown in Figure S20a,b (Supporting Information). For THF, the dQ/dV response remains nearly unchanged from the first to the 100th cycle, indicating the stability and compatibility of the as‐synthesized SE with the relatively high voltage NMC622 cathode.

The high capacity obtained for THF and ACN cells can be attributed to several factors, including the SE's high ionic conductivity and optimum morphology that ensures better contact at the cathode–SE interface and inside the cathode, and facilitates better electrochemical charge transport and minimizes the charge/discharge overpotential (Figure S19, Supporting Information).

**Figure 5:**
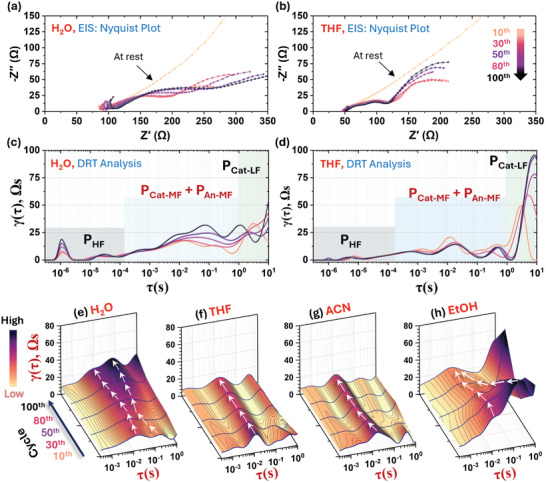
Nyquist impedance evolution of Li‐In/Li_6_PS_5_Cl//Li_3_InCl_6_/NMC622 all‐solid‐state cells during cycling at 0.2C with the Li_3_InCl_6_ SE synthesized from a) H_2_O, b) THF. c,d) Corresponding DRT analysis. The 3D DRT convolutions corresponding to the P_Cat‐MF_ region over the long‐term cycling for Li_3_InCl_6_ SE synthesized from e) H_2_O, F) THF g) ACN, and h) EtOH. All data shown in this figure are from room temperature (22 °C) cells. The component assignment has been discussed in the Supporting Information.

An insight into the cells’ interfacial contact and stability is revealed by the impedance evolution as a function of cycling, as presented in **Figures** **5**a–d and S22a–d (Supporting Information). From the Nyquist profile (Figure 5a,b; Figure S22a,b, Supporting Information), it is apparent that ACN (60 Ω) and THF (50 Ω) cells show considerably lower resistance to ion transport in the SE layer – represented by the high‐frequency intercept on Z′(Ω) axis – compared to H_2_O (90 Ω) and EtOH (100 Ω). The mid (kHz‐Hz) frequency region corresponding to the cathode‐SE or cathode‐electrolyte interface (CEI)^[^ ^40^ ^]^ reveals a much less resistive (overall resistance: 70–80 Ω) and very stable CEI for the THF and ACN cells in comparison to that for H_2_O (>150 Ω). These values were obtained by fitting the Nyquist data with the model circuit shown in Figure S21 (Supporting Information), but a full treatment as a function of cycling was avoided as the quality of fitting was not consistent.

Deconvolution of the impedance data from the frequency to time domain, which yields a distribution of relaxation time (DRT) diagram to differentiate physicochemical processes or interactions by their contributory size/area, denoted by γ(τ) and time constant in the relaxation time domain, represented by τ, depicts a more clear distinction between the cells (Figure 5c,d; Figure S22c,d, Supporting Information). The corresponding 3D plots (Figure 5e–h; Figure S22e–h, Supporting Information) highlight the evolution of the CEI region, denoted by P_Cat‐MF_ (see Figure S23 and corresponding discussion, Supporting Information). THF and ACN cells exhibit the lowest γ(τ) contribution area and minimal variation over long‐term cycling, signifying a less resistive and stable CEI. The narrow distribution width of γ(τ) further indicates a more uniform CEI for THF and ACN. In contrast, the H_2_O and EtOH cell data show a much broader distribution (Figure 5e,h). Here, the γ(τ) also varies significantly with cycling, with an increasing trend for H_2_O and a decreasing trend for EtOH for the resistive contribution from P_Cat‐MF_. Yet, even after 100 cycles, the P_Cat‐MF_ component for the EtOH cell stays higher and broader than THF and ACN. While the reason behind the observed decreasing trend for the EtOH cell is not apparent, it can likely be ascribed to the combined effect of high SE microstrain and large NMC‐SE interfacial area (small SE crystallite and particle size), which render not only enhanced interfacial degradation but also its complex evolution with cycling. The stable and uniform interface for ACN and THF can likely be ascribed to a more compact and pore‐free interface thanks to the large and agglomerated SE particles. On the other hand, finer SE particles lead to an imperfect interface that undergoes microstructural changes with cycling. Overall, the THF‐derived Li_3_InCl_6_ SE furnishes the most durable and least resistive interfaces, which explains the observed high specific capacity and excellent cycling stability.

**Figure 6:**
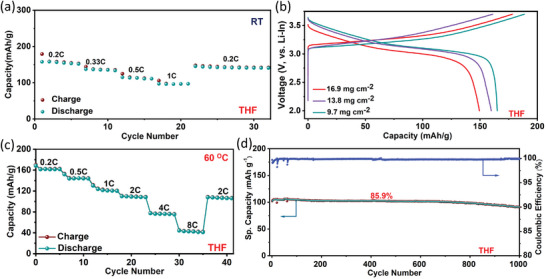
a) Rate capability of Li‐In/Li_6_PS_5_Cl//Li_3_InCl_6_/NMC622 cell with the Li_3_InCl_6_ SE derived using THF. b) The first charge/discharge curve of the solid‐state cells with increasing NMC622 loading for the THF‐derived Li_3_InCl_6_ SE. (Data shown in these two figures are from room temperature (22 °C) cells). c) Rate capability, and d) long‐term cycling stability at 2 C current rate for High‐temperature (60 °C) Li‐In/Li_6_PS_5_Cl/Li_3_InCl_6_/NMC622 cell with the Li_3_InCl_6_ SE derived using.

Given the all‐around beneficial electrochemical properties of the THF‐derived SE, the performance of the THF cell was evaluated further. **Figures** **6**a and S24a (Supporting Information) demonstrate the rate capability, with a 1 C rate capacity of 100 mAh g^−1^ and recovery of the full capacity on reversal of the current rate. Additionally, the performance of the cell was also evaluated with increased cathode loading/thickness. As shown in Figure 6b and Figure S24b (Supporting Information), increasing the active NMC622 loading from 9.7 to 13.8 and 16.9 mg cm^−2^ dropped the discharge capacity only slightly from 164 mAh g^−1^ (1.55 mAh cm^−2^) to 159.8 mAh g^−1^ (2.33 mAh cm^−2^) and 149.4 mAh g^−1^ (2.51mAh cm^−2^), respectively, which confirms the scalability of the cathode loading and obtainable capacity.

The THF cell with 10x scaled‐up Li_3_InCl_6_ also displays high capacity and excellent stability (Figure S25a, Supporting Information). A first cycle discharge capacity of 145 mAh g^−1^ with a stable polarization profile as a function of cycling (Figure S25b, Supporting Information) and the 97.3% capacity retention after 100 cycles with a remarkable average Coulombic efficiency of 99.75% confirms the feasibility of THF‐mediated wet‐chemical synthesis route for producing high‐quality Li_3_InCl_6_ SE for practical development.

Finally, the suitability and stability of the THF‐derived Li_3_InCl_6_ as a functional solid electrolyte was probed by high‐temperature (60 °C) testing of the solid‐state cells. Figure 6c displays the excellent rate capability data with specific capacities of 75 and 46 mAh g^−1^ at a significantly high current density of 4 C and 8 C, respectively. On a long‐term cycling test at a 2 C current rate, the cell exhibits an impressive initial discharge capacity of 105.2 mAh g^−1^ and retention of 85.9% after 1000 cycles at an average CE of 99.45% (Figure 6d). These outstanding properties are owed to higher ionic conductivity at high temperatures and, most importantly, excellent electro‐chemo‐mechanical stability of the interfaces, which is evident from Figure S26 (Supporting Information). The stability of the interfaces and the CEI is apparent from the small evolution of the impedance over 1000 cycles and a rather small, combined resistance of ≈80 Ohms in the 1000th cycle. These findings showcase the viability of the aprotic THF‐mediated wet chemical synthesis as a facile and scalable approach for the preparation of high‐quality Li_3_InCl_6_ SE for all‐solid‐state lithium battery applications.

## 3 Conclusion

In conclusion, a facile and scalable wet chemical method is demonstrated to prepare highly conducting Li_3_InCl_6_ solid electrolytes using aprotic (THF, ACN) and protic (H_2_O, EtOH) solvents. The evaporative crystallization from different solvents leads to varying microstructural characteristics, with the aprotic solvents rendering minimal microstrain and larger crystallites that are conducive to achieving high ionic conductivity. Consequently, the THF‐derived Li_3_InCl_6_ displays the record Li^+^ conductivity of 4.03 mS cm^−1^ for halide‐type SEs at room temperature (22 °C). Additionally, reduced grain boundary area for the THF‐derived halide facilitates improved stability to humidity. The optimal microstructure of the THF‐derived halide leads to the least resistive and stable interfaces in solid‐state cells assembled with NMC622 cathode, resulting in high specific capacity, good rate capability, and excellent cycling stability with high Coulombic efficiency at room temperature and 60 °C. It is shown that the developed synthetic route is easily scalable, and the scaled‐up synthesis leads to high‐performance SE, asserting the viability of the aprotic solvent‐mediated synthesis of Li_3_InCl_6_ solid electrolyte for practical development.

## Conflict of Interest

The authors declare no conflict of interest.

## Supporting information

Supporting Information

## Data Availability

The data that support the findings of this study are available from the corresponding author upon reasonable request.
