# Supplementary material for: Wet Chemistry Route to Li3InCl6: Microstructural Control Render High Ionic Conductivity and Enhanced All‐Solid‐State Battery Performance
Source: Adv Sci (Weinh). 2024 Jul 8;11(34):2403208. doi: 10.1002/advs.202403208 (PMC11425892; doi:10.1002/advs.202403208)
Supplement: Supplementary file 1 — Supporting Information [file ADVS-11-2403208-s001.pdf]

ADVANCED SCIENCE
Open Access

Supporting Information

for *Adv. Sci.*, DOI 10.1002/advs.202403208

Wet Chemistry Route to Li_3InCl_6: Microstructural Control Render High Ionic Conductivity and Enhanced All-Solid-State Battery Performance

*Jacob Otabil Bonsu, Abhirup Bhadra and Dipan Kundu**

Supporting Information

Wet Chemistry Route to Li_3InCl_6: Microstructural Control Render High Ionic Conductivity and Enhanced All-Solid-State Battery Performance

Jacob Otabil Bonsu,[a] *Abhirup Bhadra,*[a] *Dipan Kundu*[a,b,*]

[a] *School of Chemical Engineering, UNSW Sydney, Kensington, NSW 2052, Australia*

[b] *School of Mechanical and Manufacturing Engineering, UNSW Sydney, Kensington, NSW 2052, Australia*

**Corresponding author. Email: d.kundu@unsw.edu.au*

Experimental Methods

Synthesis of Li_3InCl_6 solid electrolyte via solvent-mediated route. A stoichiometric ratio (3:1) of indium chloride ($InCl_3$, Sigma-Aldrich, 99.9%) and lithium chloride (LiCl, Sigma-Aldrich, 99.9%) precursors were weighed and dissolved in the desired solvent (deionized water; acetonitrile/ethanol/tetrahydrofuran, analytical reagents, Sigma-Aldrich). This initial process was conducted in an ambient environment. The solutions were evaporated at room temperature (22°C) and made to crystallize without applying any heat. They were then dried in a vacuum oven at 80°C to rid the crystallized intermediate of any solvent. Finally, the dried precursors were pulverized and heat-treated at 200°C under vacuum to obtain pristine Li_3InCl_6 solid electrolytes. 10x scaled Li_3InCl_6 electrolytes were synthesized via the same approach but with vamped-up stoichiometric molar ratios. Samples synthesized are identified according to the solvent in which they were synthesized (i.e., THF, ACN, EtOH, H_2O)

Conductivity measurements

The ionic and electronic conductivities of the pristine and 10x scaled-up Li_3InCl_6 solid-electrolytes were measured by AC impedance spectroscopy and DC polarisation test, respectively. Electrochemical impedance spectroscopy (EIS) measurement was conducted on a cold-pressed pellet (pressed at 310 MPa without any heat treatment) in the 1 MHz - 100 mHz range by applying a 50 mV signal perturbation on a BioLogic VMP-3e Potentiostat. The pellet, with an estimated thickness of 1.08 mm, was sandwiched between two steel plungers in a

homemade cell with a peek body (die) of 12 mm internal diameter. The same cell setup was used for the DC polarization measurement for the electronic conductivity evaluation, and different voltage biases (0.1 to 0.5 V) were applied for 3 hours to reach a steady current response.

Characterisation

The crystal structure and phase purity of the samples were examined using X-ray diffraction (XRD). The diffraction patterns (2θ from 10° to 80°) were obtained on Panalytical Aeris diffractometer with copper Kα radiation (λ = 1.54178 Å), at an optimal scan rate of 2 degrees per minute, using an air-tight sample holder to prevent air exposure. Thermo Scientific K-Alpha+ X-ray photoelectron spectrometer with Al Kα X-ray was employed for X-ray photoelectron spectroscopy (XPS) analysis. A Hitachi S-4800 field emission scanning electron microscopy (SEM) was used to characterize the morphologies and estimate the particle size of the wet chemical synthesized Li_3InCl_6.

Electrochemical Studies

The all-solid-state lithium batteries (ASSLBs) were fabricated inside an argon-filled glovebox maintained at H_2O < 0.1 ppm and O_2 < 1 ppm. An in-house designed cell (die internal diameter: diameter 12 mm) setup capable of applying the desired/fixed assembly and stack pressure was used.[1] For Li_xIn (x ~ 0.5) anode (negative electrode) preparation, lithium and indium metal foils were pressed and rolled for 15 minutes and rested for 24 hours. The cathode (positive electrode) composite mixture was prepared by hand grinding commercial $LiNi_{0.6}Mn_{0.2}Co_{0.2}O_2$ (NMC-622, MSE Supplies LLC, AZ, USA), and the wet chemical synthesized Li_3InCl_6 solid-state electrolyte in a 70:30 weight ratio for 20 minutes. For cell preparation, 30 mg of Li_6PS_5Cl solid electrolyte powder (Ampcera®, MSE Supplies LLC, AZ, USA) was pressed at 126 MPa to form a thin layer. 70 mg of the wet chemical synthesized Li_3InCl_6 SSE was spread uniformly on the thin sulfide layer and pressed at 150 MPa. Then ~22 mg of cathode composite powder was evenly spread on the Li_3InCl_6 side and pressed at 512 MPa. Finally, the Li_xIn alloy anode disc was placed on the sulfide electrolyte side to complete the electrode stack. Anode and cathode plungers fitted with O rings ensured hermetic sealing during cell cycling. Cells were removed from the glove box, placed under an in-house designed compression jig,[1,2] and electrochemically cycled under a steady uniaxial stack pressure of 45 MPa. The cells were galvanostatically cycled on a Land cycler in the 2 - 3.7 V (*vs*. Li_xIn) window.

Distribution of Relaxation Time (DRT) Analysis

Due to the complexities associated with Nyquist plots, we utilized deconvolution of Electrochemical Impedance Spectroscopy (EIS) data from frequency to time domain, yielding a Distribution of Relaxation Time (DRT) diagram to differentiate physicochemical processes or interactions by their contributory size/ area, denoted by $\gamma(\tau)$ and time constant in the relaxation time domain, represented by τ. (**Figure 5c-d, S20c-d**)

We utilized the raw data collected from EC lab software with appropriate formatting as the input files for the Python-based **pyDRTtools software**, which is available as open source by the Ciucci lab. The primary objective of the DRT is to ascertain the distinctive distribution of standard EIS timescales. To achieve this, the experimental data Z_{exp}, measured at specific frequencies, are compared to a model Z_{DRT} derived from the subsequent expression.

$$Z_{DRT}(f) = R_{\infty} + \int_{0}^{\infty} \frac{g(\tau)}{2\pi f\tau} d\tau$$

where R_{∞} is the ohmic resistance, and $g(\tau)$ is a suitable function that describes the time relaxation characteristics of the electrochemical system studied. Expression (1) can be interpreted as an equivalent Voigt circuit. Put simply, the impedance model of the DRT consists of an Ohmic resistance (at $f \rightarrow \infty$) along with summation of infinite series of parallel resistors with capacitors (RC circuits). Since the Frequency in Impedance dta collection is l logarithmically spaced the logarithmic correlation of $g(\tau)$ can be established with τ with the following equation:

$$Z_{DRT}(f) = R_{\infty} + \int_{0}^{\infty} \frac{\gamma(ln(\tau))}{2\pi f\tau} dln\,\tau$$

In the software we used Combine Z_{Re} and Z_{Im} data with inductance data being discarded during fitting. For discretization the Gaussian radial basis functions (RBF) were used with FWHM based control of shape. with regularization method controlled by GCV function and regularization parameter at 0.001 for second order regularization derivative. For further detailed treatment method the relevant literature is cited here. [3,4]

Based on prior literature on DRT plot analysis for all-solid-state batteries, our Li_3InCl_6 catholyte-based cells have been assigned three major contributory regions: P_{HF}, $P_{Cat\text{-}MF}$, and P_{LF}. Due to the limitations in data collection at low-frequency domains (frequency cutoff at 10 mHz), P_{LF}, primarily representing ion diffusion in solid state, is excluded from comparison.

Furthermore, P_{HF}, attributed to the anode and anode-SE interface processes, is excluded as Li_6PS_5Cl anolyte and Li-In anode were constant across the control variations. The middle-frequency regions, representing cathode-solid electrolyte interactions (indicated as P_{Cat-MF}), primarily lie in the range of 10^{-4} s to 1s (in the order of 1 Hz to few kHz, in frequency domain).[5]

Crystallite size and microstrain from the Williamson-Hall method

In the Williamson-Hall analysis, XRD peak broadening is considered to predominantly arise due to the collective influence of microstrain and crystallite size, i.e.,[6,7]

Total broadening = broadening due to strain + broadening due to crystallite size

$$\beta_T = \beta_\varepsilon + \beta_D \qquad (1)$$

Where total broadening is β_T, broadening due to the microstrain is β_ε and the broadening due to crystallite size is β_D.

From the Scherrer equation in X-ray diffraction and crystallography, which relates crystallite size with XRD peak broadening, we have:

$$D = \frac{K\lambda}{\beta_D cos\theta} \qquad (2)$$

$$\text{Or, } \beta_D = \frac{K\lambda}{D cos\theta} \qquad (3)$$

Where β_D is peak broadening due to crystallite size or full width at half maximum (FWHM) in radian, D is the crystallite size, λ is the wavelength of the X-ray source (λ = 0.15405 nm), K is the dimensionless shape factor, expressed as 0.9 ($K = 2\sqrt{(ln2)/\pi}$), θ is the peak position in radian.

Therefore, the XRD peak broadening due to the microstain can be expressed as

$$\beta_\varepsilon = 4\varepsilon tan\theta \qquad (4)$$

Where β_ε is broadening due to microstrain, θ is the peak position in radian, and ε is the strain.

Substituting equations (4) and (3) in equation (2), yields:

$$\beta_T = \frac{K\lambda}{D cos\theta} + 4\varepsilon tan\theta \qquad (5)$$

From Trigonometric functions where, $tan\theta = \frac{sin\theta}{cos\theta}$ and substituting this into equation 4, we arrive at:

$$\beta_T = \frac{K\lambda}{D cos\theta} + \frac{4\varepsilon sin\theta}{cos\theta} \quad (6)$$

Rearranging equation 5 yields,

$$\beta_T cos\theta = \varepsilon(4sin\theta) + \frac{K\lambda}{D} \quad (7)$$

Considering the standard equation of a straight line i.e.

$y = mx + c$,

Equation (6) is treated as a straight-line equation, in which $\beta_T cos\theta$ is the ordinate (y- axis), ε is the gradient of the line, $4sin\theta$ is the abscissa (x-axis), and $\frac{K\lambda}{D}$ is the intercept on the y-axis.

Consequently, using $4sin\theta$ as x-axis and $\beta_T cos\theta$ as y-axis to generate the Williamson-Hall plot aids in calculating the microstrain(ε) and crystallite size (D) of the samples.

The standard crystalline sample was used for the position and instrumental broadening calibration to prevent the impact of instrumental broadening.

Table S1. Comparative study of Li_3InCl_6 halide solid electrolyte synthesized by mechanical milling method and Solvent mediated route.

**Halide SSE**	**Synthesis Method**	**mS/cm (Ionic Conductivity)**	**Morphology & particle size**	**Crystal Structure**	**Refs.**
Li_3InCl_6 SE	Ball Milling	0.84 @ 25°C	Nanoparticles ~ 200 nm	Monoclinic *C2/m*	[8]
Li_3InCl_6 SE	Ball Milling	1.49 @ 25°C	Nanoparticles ~500 nm	Monoclinic *C2/m*	[8]
Li_3InCl_6 SE	Ball Milling	0.98 @ 25°C	Nanocrystalline	Monoclinic *C2/m*	[9]
Li_3InCl_6 SE	Ball Milling	1.03 @ 25°C	Micro sized	Monoclinic *C2/m*	[10]
Li_3InCl_6 SE	Ball Milling	>1.0 @ 25°C	Nanoparticles 78.5% ~ 600nm	Monoclinic *C2/m*	[11]
Li_3InCl_6 SE	Water mediated	>1.5 @ 25°C	Microparticles 78.5% ~ 1µm	Monoclinic *C2/m*	[11]
Li_3InCl_6 SE	Water (HCl assisted) mediated	1.94 @ 25°C	Micron-sized ~ 10 µm	Monoclinic *C2/m*	[12]

Li_3InCl_6 SE	Water mediated	2.04 @ 25°C	Micron-sized ~ 1µm	Monoclinic *C2/m*	[13]
Li_3InCl_6 SE	H_2O mediated (freeze dry)	>2.0 @ 25°C	Nanoparticles 80% ~ 200 nm	Monoclinic *C2/m*	[11]
Li_3InCl_6 SE	Ethanol mediated	0.79 @ 25°C	Micromorphology ~2-5 µm	Monoclinic *C2/m*	[14]
Li_3InCl_6 SE	H_2O mediated	2.72@ 25°C	Micron-sized ~2-5 µm	Monoclinic *C2/m*	**This work**
Li_3InCl_6 SE	EtOH mediated	3.07@ 25°C	Micron-sized ~3-7 µm	Monoclinic *C2/m*	**This work**
Li_3InCl_6 SE	ACN mediated	3.67@ 25°C	Micron-sized ~10-20 µm	Monoclinic *C2/m*	**This work**
Li_3InCl_6 SE	THF mediated	4.03@ 25°C	Micron-sized ~10-30 µm	Monoclinic *C2/m*	**This work**

Table S2. Pristine Li_3InCl_6 ionic conductivity values.

**Sample**	**Temperature (°C)**	**Ionic Conductivity (mS/cm)**
H_2O	RT (22)	2.72
EtOH	RT (22)	3.07
ACN	RT (22)	3.67
THF	RT (22)	4.03
H_2O	35	7.73
EtOH	35	7.78
ACN	35	11.23
THF	35	14.30
H_2O	45	9.47
EtOH	45	9.85
ACN	45	13.62
THF	45	16.87

H_2O	55	10.72
EtOH	55	11.21
ACN	55	17.18
THF	55	20.98
H_2O	65	13.90
EtOH	65	15.12
ACN	65	23.06
THF	65	25.50
H_2O	75	17.16
EtOH	75	19.52
ACN	75	31.85
THF	75	33.92
H_2O	85	21.64
EtOH	85	24.71
ACN	85	37.55
THF	85	38.46

Table S3. 10x scaled-up Li_3InCl_6 ionic conductivity values

**Sample**	**Temperature (^oC)**	**Ionic Conductivity (mS/cm)**
H_2O	RT (22)	2.08
EtOH	RT (22)	2.35
ACN	RT (22)	3.13
THF	RT (22)	3.67
H_2O	35	2.50
EtOH	35	3.05
ACN	35	6.02

THF	35	6.22
H_2O	45	2.82
EtOH	45	3.42
ACN	45	10.17
THF	45	14.52
H_2O	55	3.81
EtOH	55	4.40
ACN	55	12.35
THF	55	19.47
H_2O	65	5.17
EtOH	65	6.75
ACN	65	18.90
THF	65	24.71
H_2O	75	5.91
EtOH	75	8.53
ACN	75	24.83
THF	75	31.72

Table S4. Ionic conductivity degradation after 10% humidity exposure at room temperature (22°C)

**Sample**	**Duration (Hours)**	**Ionic Conductivity (S/cm)**
H_2O	24	$2.34*10^{-4}$
EtOH	24	$7.51*10^{-4}$
ACN	24	$8.14*10^{-4}$
THF	24	$9.14*10^{-4}$
H_2O	48	$1.35*10^{-4}$

EtOH	48	$1.85*10^{-4}$
ACN	48	$2.33*10^{-4}$
THF	48	$4.76*10^{-4}$
H_2O	72	$7.18*10^{-5}$
EtOH	72	$8.95*10^{-5}$
ACN	72	$1.07*10^{-4}$
THF	72	$2.83*10^{-4}$

Table S5. Ionic conductivity data at room temperature after 50% humidity exposure data at room temperature

**Sample**	**Duration (Hours)**	**Ionic Conductivity (S/cm)**
H_2O	24	$1.91*10^{-6}$
EtOH	24	$2.5*10^{-6}$
ACN	24	$3.64*10^{-6}$
THF	24	$1.19*10^{-5}$

Table S6. Impedance values from equivalent circuit fitting for Li_3InCl_6/NMC622 (cathode/SE mixture) on (a) 0th day and (b) 10th day.

**0th Day**				
**$Z^I(\Omega)$**	**H_2O**	**EtOH**	**ACN**	**THF**
**R1**	10.42	12.35	9.02	9.15
**R2**	54.07	33.64	14.73	8.78
**R3**	11.68	3.58	1.25	0.45
**10th Day**				
**$Z^I(\Omega)$**	**H_2O**	**EtOH**	**ACN**	**THF**
**R1**	28.85	22.01	11.84	12.23
**R2**	57.94	77.81	19.78	8.99
**R3**	1.52	0.65	0.42	0.34

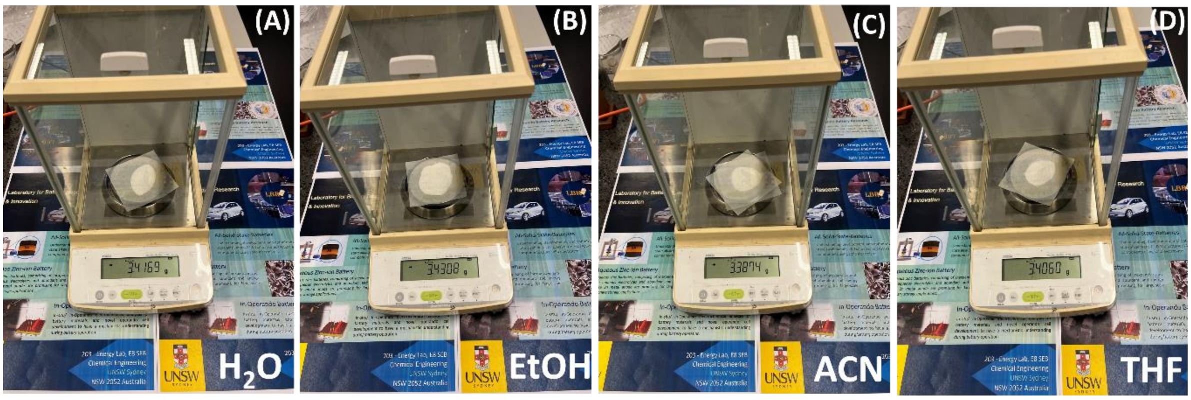

Figure S1. Representative photographs of 10x scaled-up synthesis of Li_3InCl_6 SSEs.

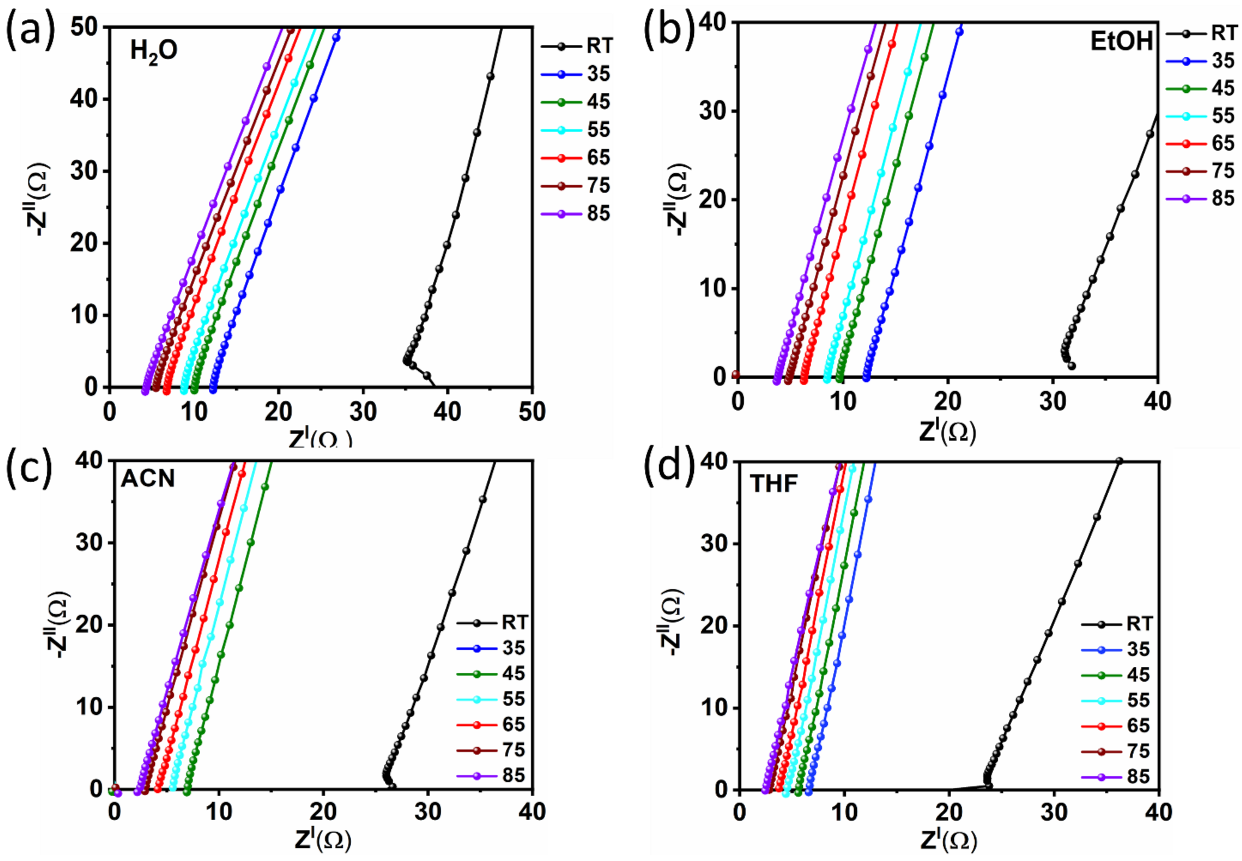

Figure S2. Temperature-dependent Nyquist impedance plots of Li_3InCl_6 SEs synthesized from (a) H_2O, (b) EtOH, (c) ACN, and (d) THF.

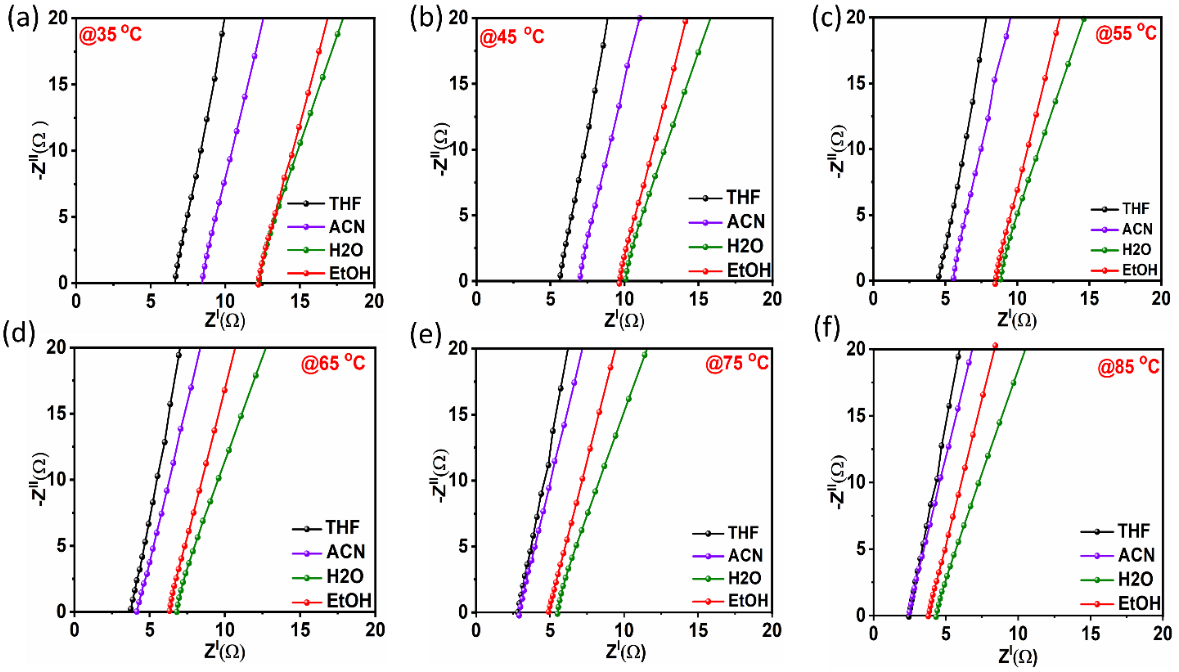

Figure S3. (a-f) Comparative Nyquist impedance plots for wet chemical synthesized pristine Li_3InCl_6 SSEs at different temperatures.

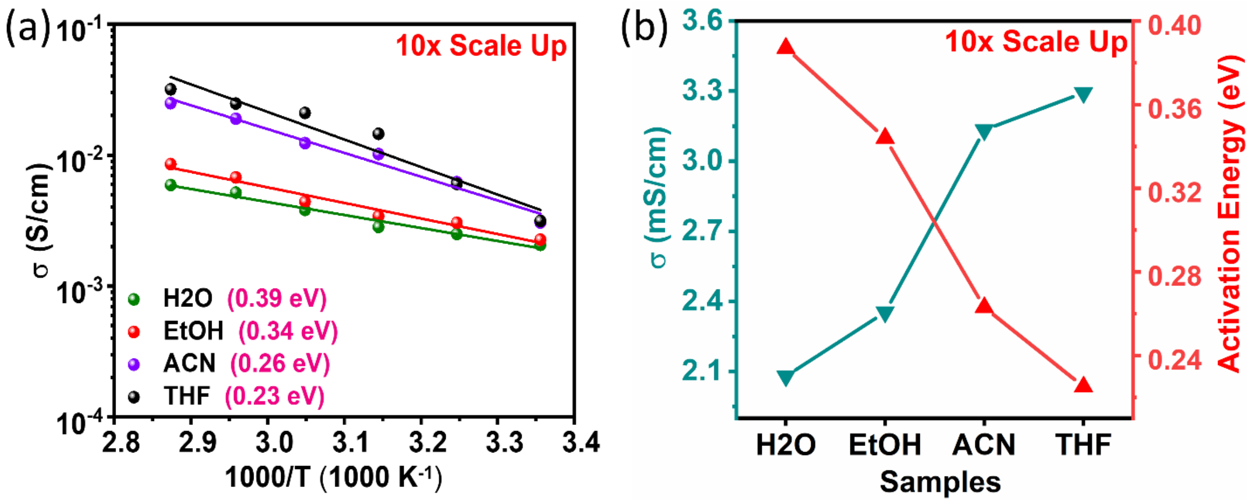

Figure S4. (a) Arrhenius fitting of the temperature-dependent ionic conductivity data for 10x scaled-up Li_3InCl_6 SEs. (b) Room Temperature ionic conductivities and activation energies of scaled-up Li_3InCl_6 SE samples.

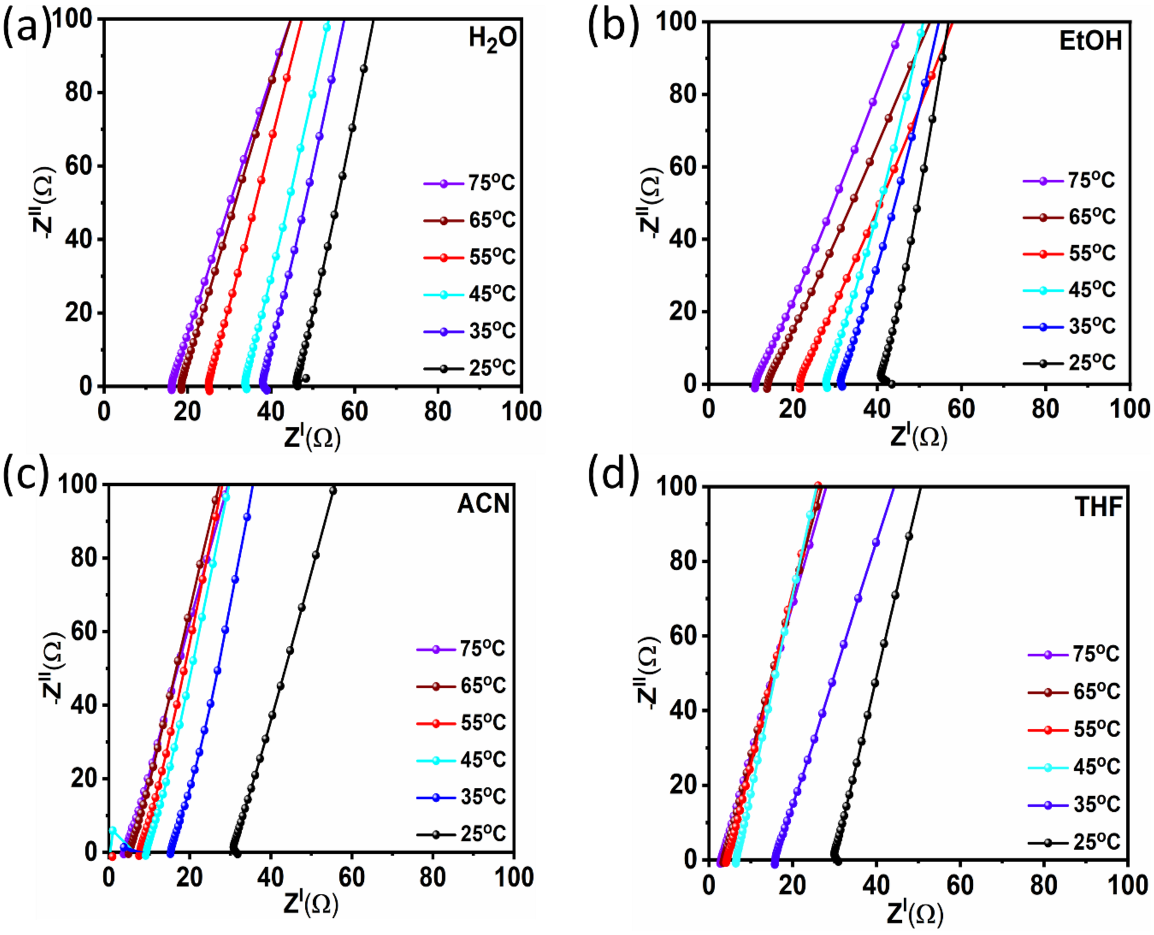

Figure S5. Temperature-dependent Nyquist impedance plots for the 10x scaled-up Li_3InCl_6 SEs synthesized from (a) H_2O (b) EtOH, (c) ACN, and (d) THF.

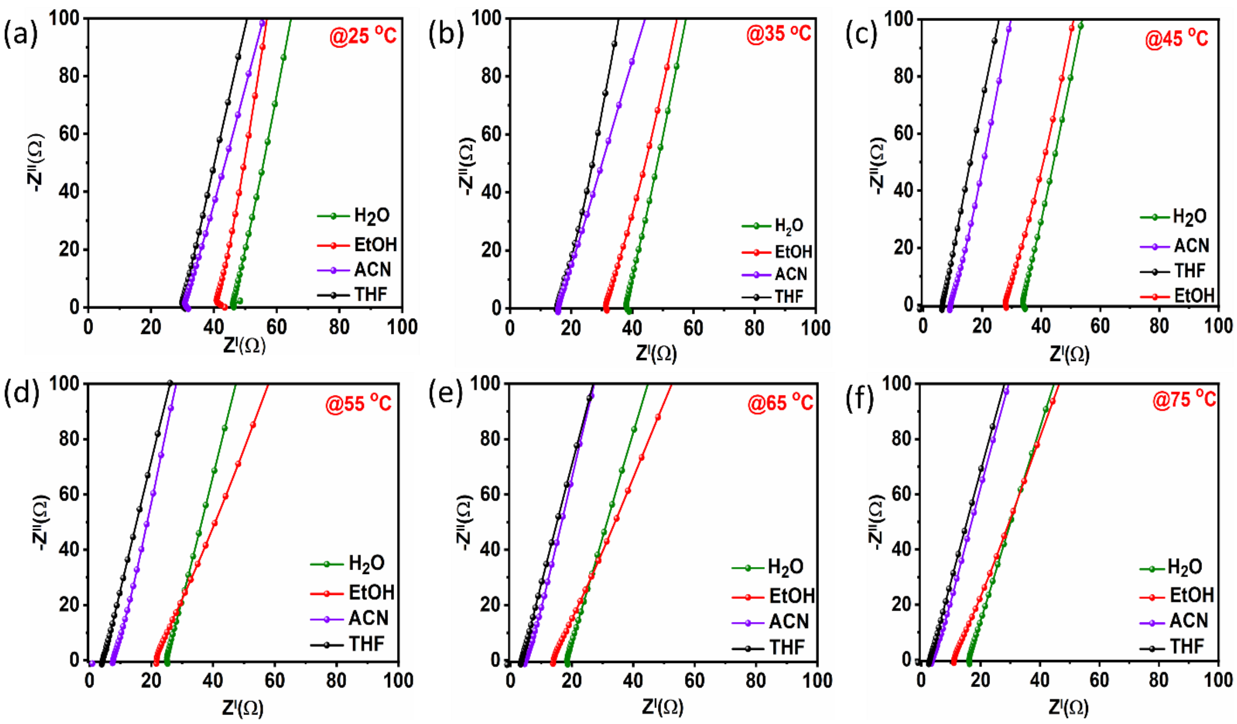

Figure S6. (a-f) Comparative Nyquist impedance plots for wet chemical synthesized 10x scaled-up Li_3InCl_6 SSEs at different temperatures.

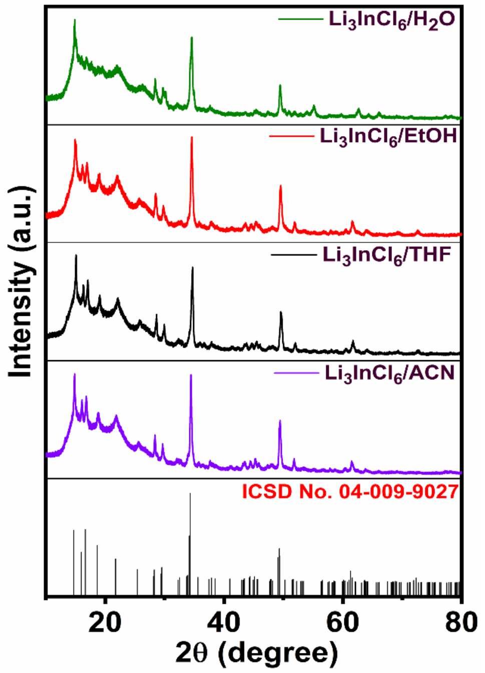

Figure S7. Slow-scanned powder X-ray diffraction patterns of 10X scaled-up Li_3InCl_6 SEs prepared by the wet chemical synthesis method

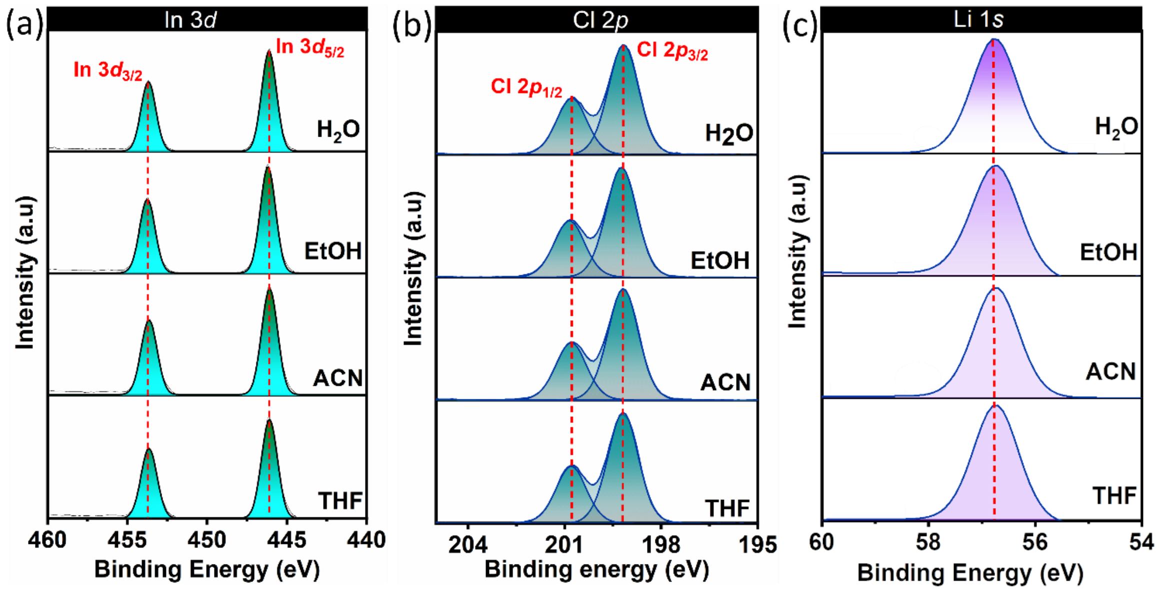

Figure S8. (a) In 3*d*, (b) Cl 2*p*, and (c) Li 1*s* X-ray photoelectron spectra for pristine Li_3InCl_6 SEs synthesized by the wet chemical method using different solvents. In $3d_{3/2}$ and $3d_{5/2}$ peaks (453.5 and 446.13 eV), Cl $2p_{1/2}$ and Cl $2p_{3/2}$ peaks (200.78 and 199.17 eV), and Li 1*s* peaks (56.76 eV) do not exhibit any noticeable shift across all four samples. Moreover, peaks corresponding to other oxidized or hydrolyzed species are also not observed, confirming the absence of any discernable impurity.

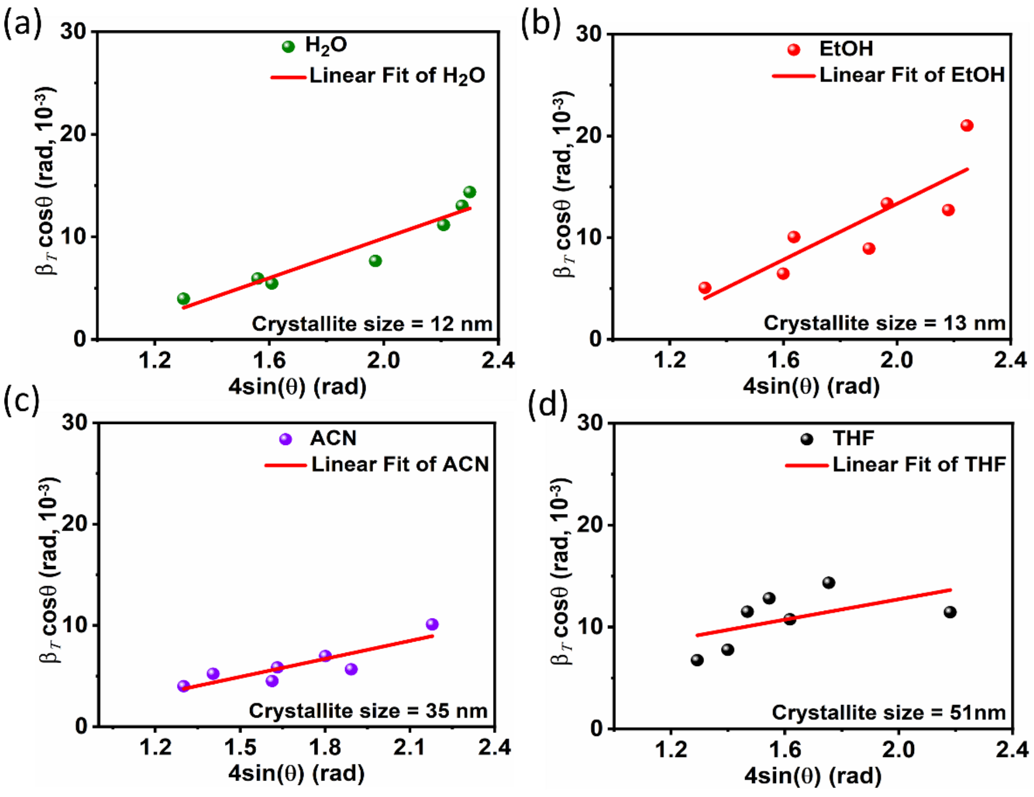

Figure S9. Williamson-Hall plot for the 10x scaled-up Li_3InCl_6 SSEs, synthesized from (a) H_2O, (b) EtOH, (c) ACN, and (d) THF.

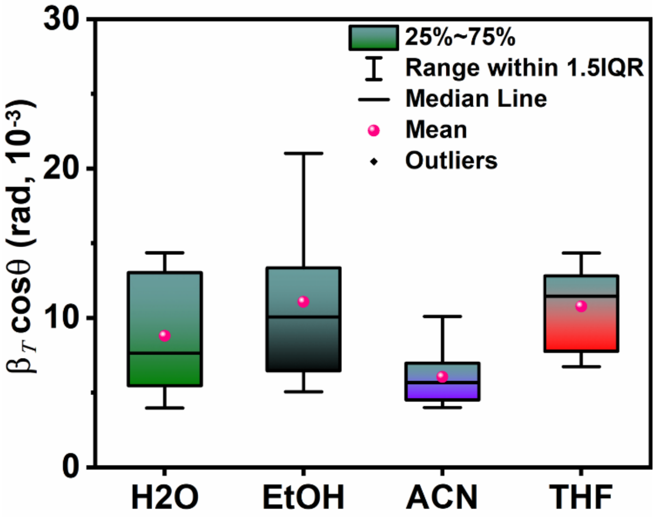

Figure S10. Box plot of the FWHM values of the XRD peaks for the 10x scaled-up Li_3InCl_6 SEs.

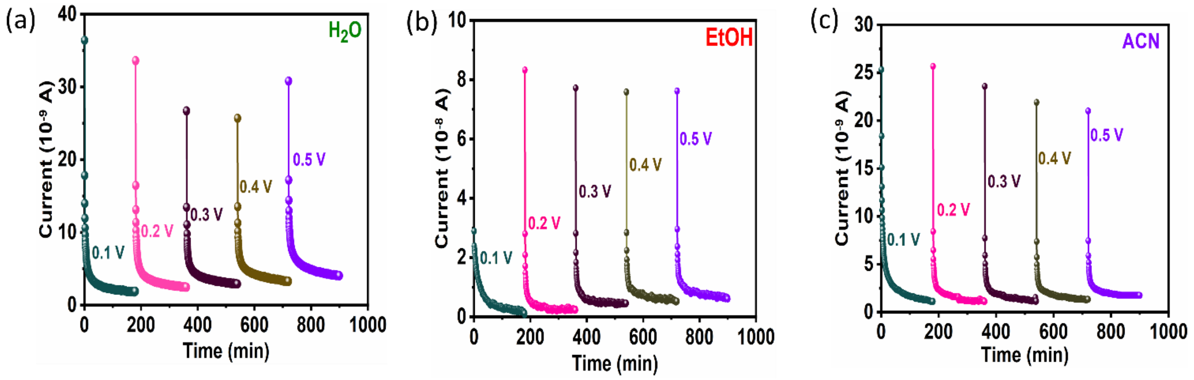

Figure S11. DC polarization curves for the as-synthesized Li_3InCl_6 SEs obtained using (a) H_2O, (b) EtOH, and (c) ACN by applying different voltage biases from 0.1 to 0.5 V in symmetric blocking electrode configuration.

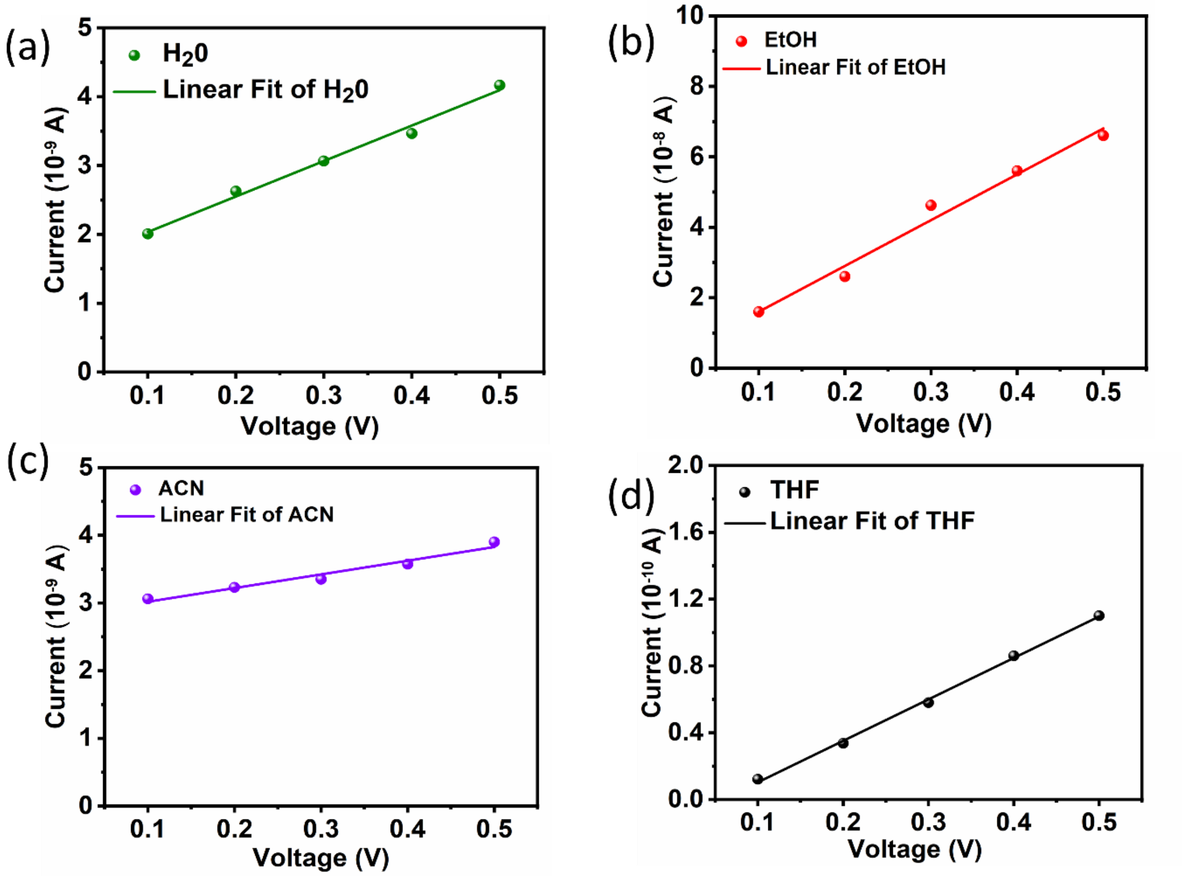

Figure S12. Equilibrium current response of symmetric cells at different voltages for pristine Li_3InCl_6 SEs synthesized using (a)H_2O, (b) EtOH, (c) ACN, and (d) THF were probed. Electronic conductivity is calculated by a similar method used for estimating the ionic conductivity. Here, the resistance values incorporated in the conductivity equation is calculated from the slope of the fitted equilibrium current response of symmetric cells at different voltages.

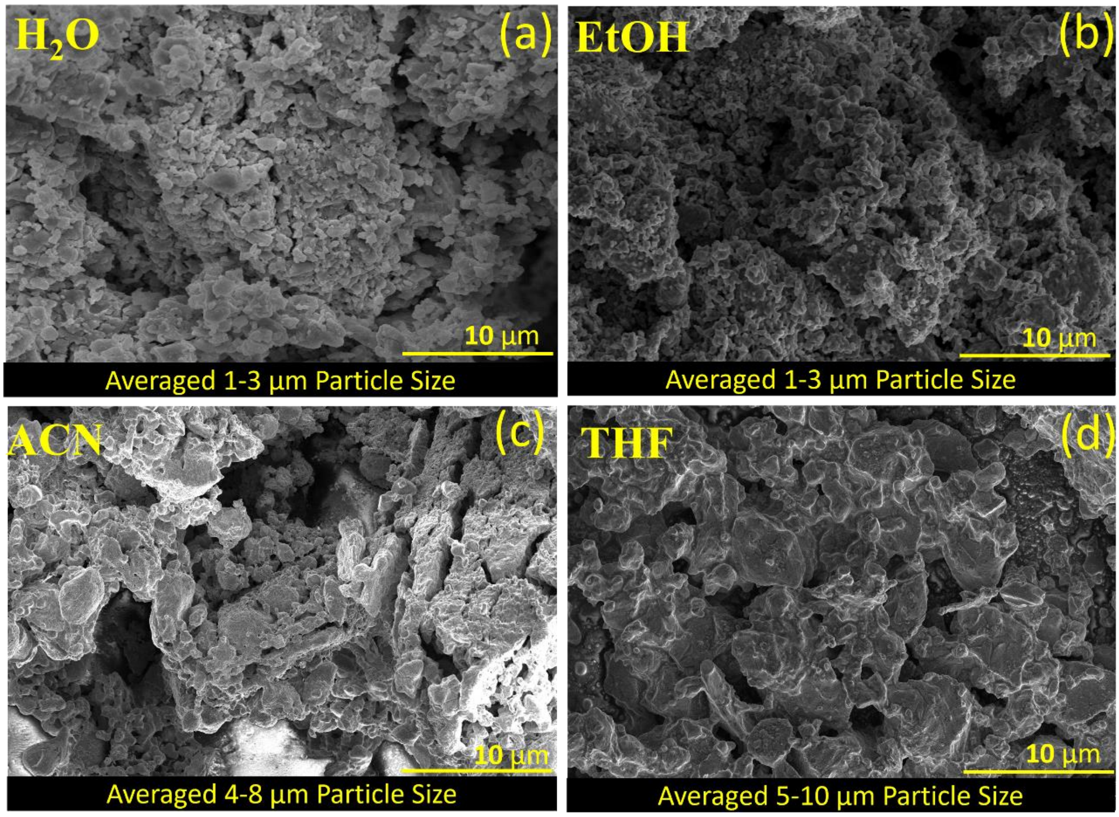

Figure S13. Representative SEM images of 10x scaled-up Li_3InCl_6 SEs synthesized using different solvents - (a) H_2O, (b) EtOH, (c) ACN, and (d) THF.

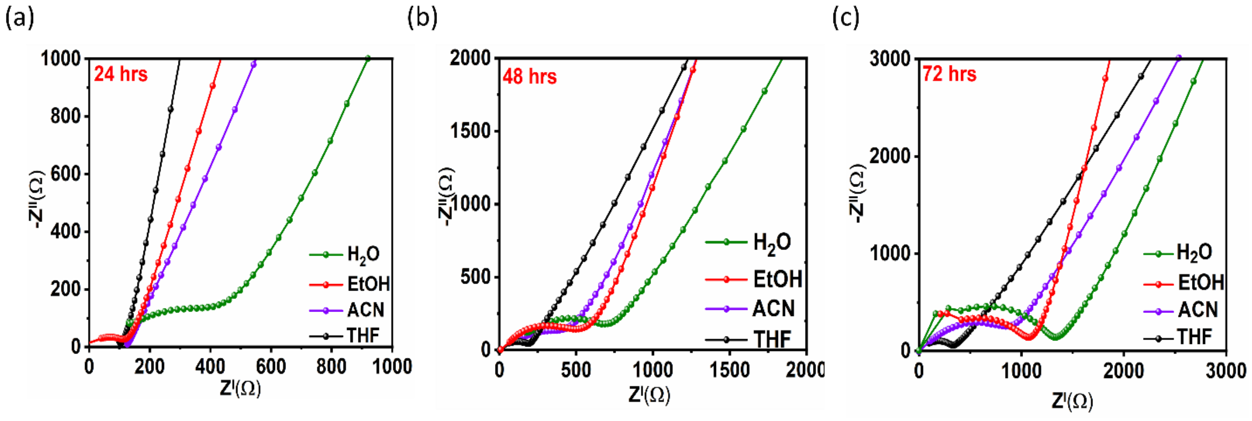

Figure S14. Nyquist impedance plots for the Li_3InCl_6 SEs after being exposed to a 10% humidity environment for (a) 24 hours, (b) 48 hours, and (c) 72 hours.

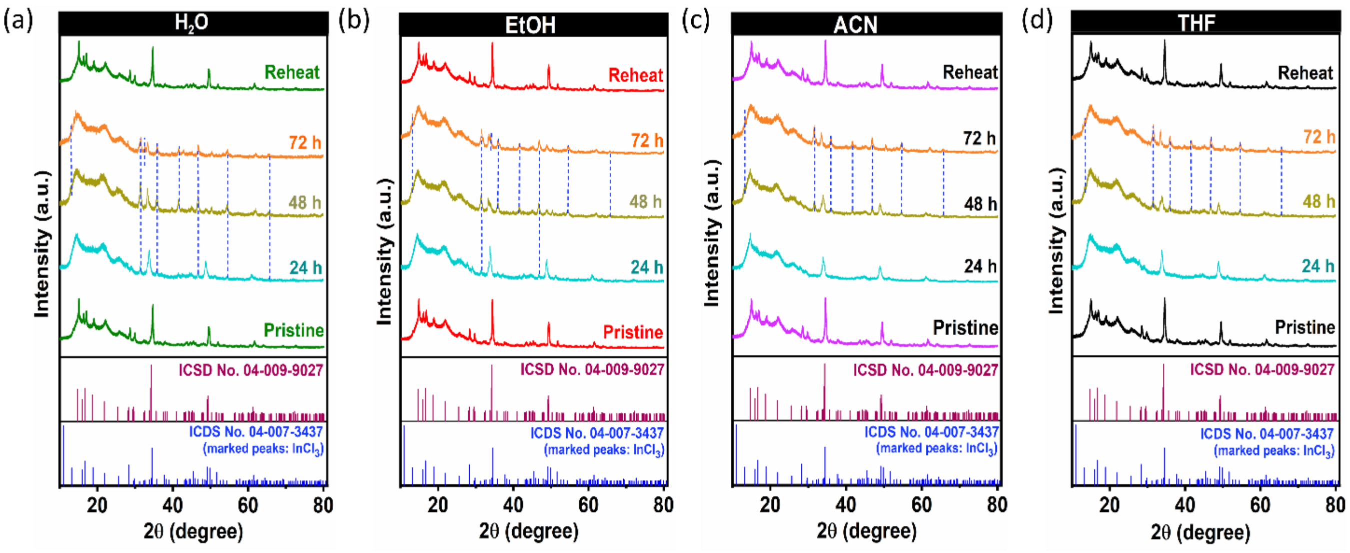

Figure S15. The XRD pattern evolution of Li_3InCl_6 SEs synthesized using (a) EtOH, (b) H_2O, (c) ACN, and (d) THF after exposure to 10% humidity condition for different durations. The plots also show the XRD pattern of the reheated SEs.

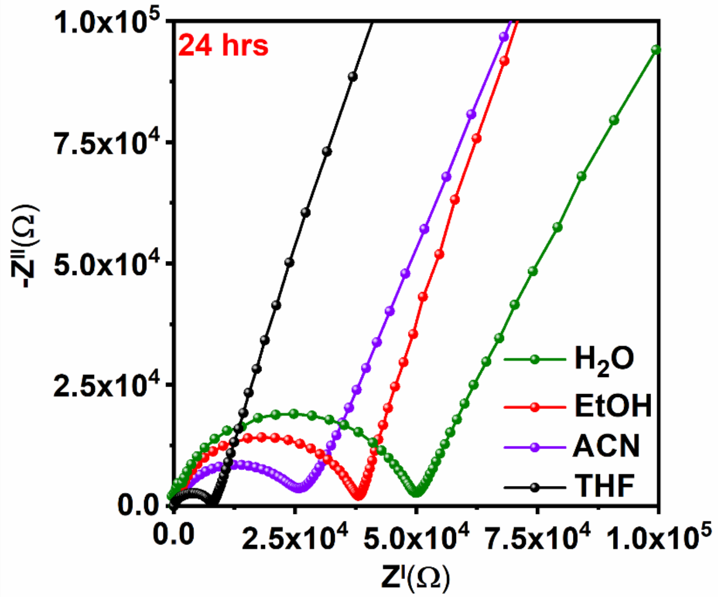

Figure S16. Nyquist impedance plots of the Li_3InCl_6 SE samples exposed to a 50% humidity environment for 24 hours.

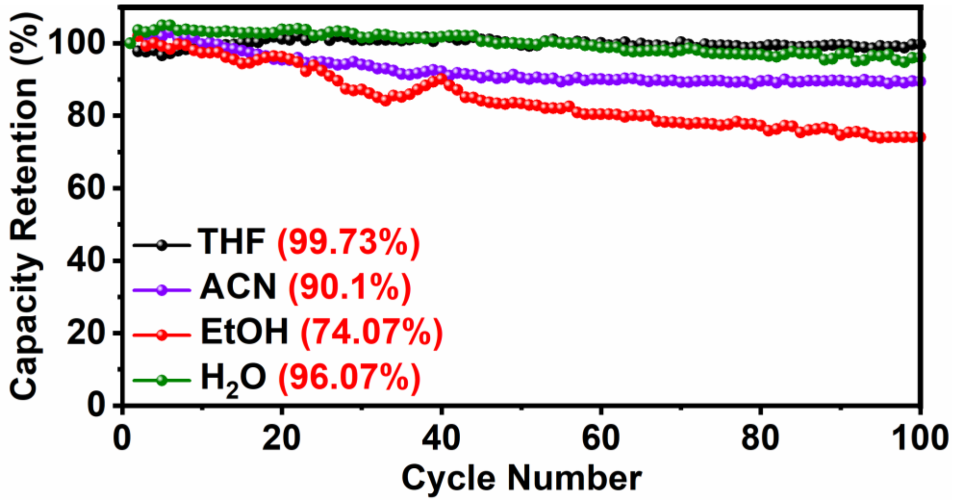

Figure S17. Comparison of the capacity retention for Li-In|li$_6$PS$_5$Cl|Li$_3$InCl$_6$/NMC622 all-solid-state cells with Li$_3$InCl$_6$ SEs obtained by the wet chemical route using different solvents. The cells were cycled at 0.2C at room temperature (22°C).

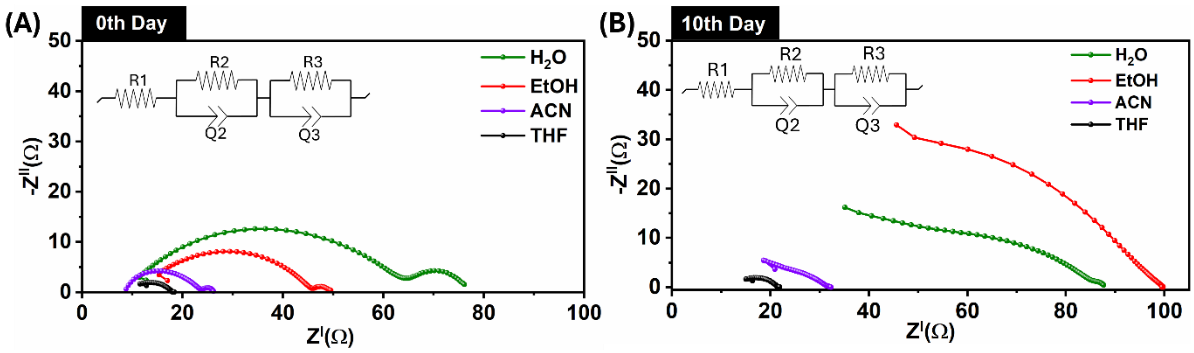

Figure S18. Nyquist impedance plots for Li$_3$InCl$_6$/NMC622 (cathode/SE mixture) on (a) 0th day and (b) 10th day.

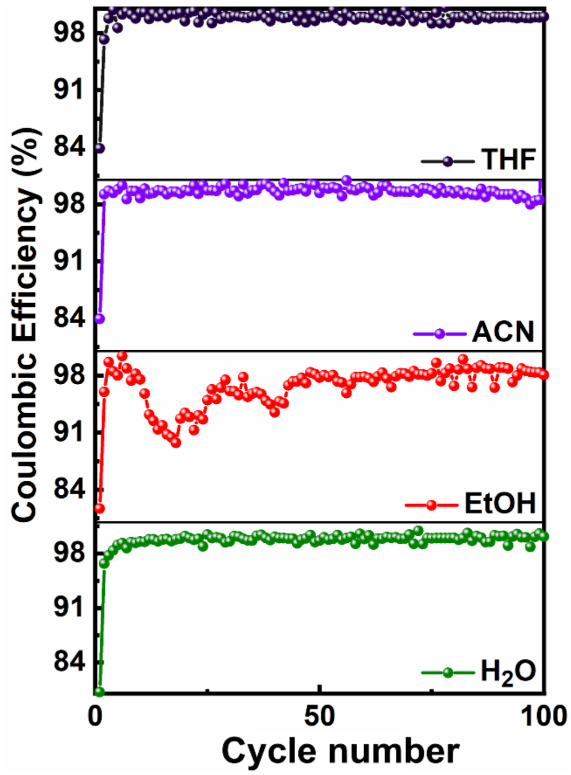

Figure S19. Coulombic efficiency plot for Li-In|Li_6PS_5Cl||Li_3InCl_6|NMC622 solid-state cells with Li_3InCl_6 SEs synthesized by the wet chemical method. The cells were cycled at 0.2C at room temperature (22°C).

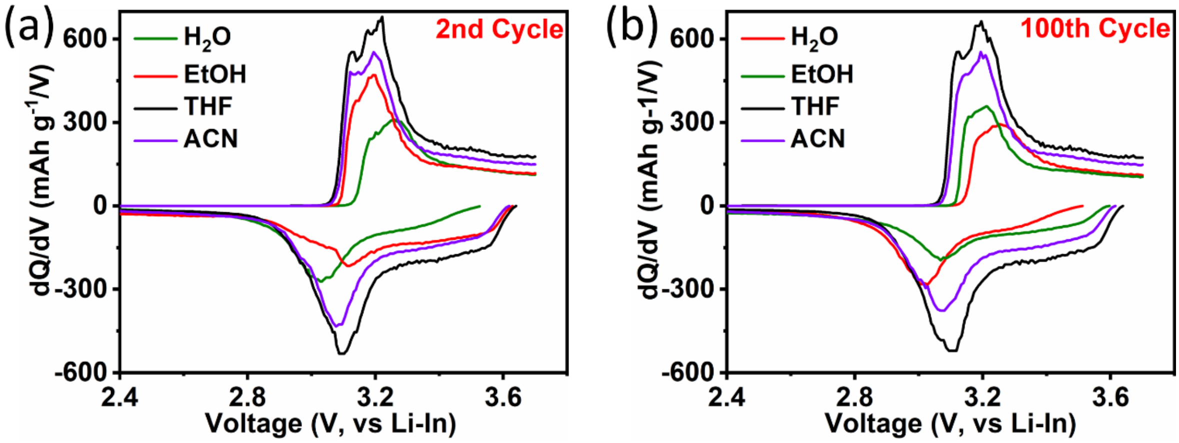

Figure S20. Differential capacity (dQ/dV) plots for Li-In|Li_6PS_5Cl||Li_3InCl_6|NMC622 all-solid-state cells with wet chemical route derived Li_3InCl_6 SEs for the (a) 2nd and (b) 100th cycle.

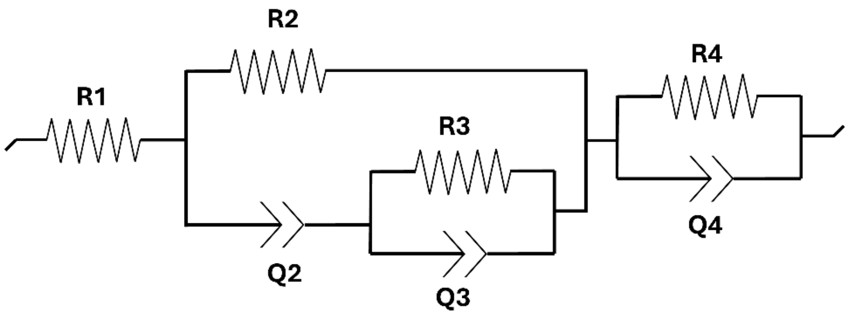

Figure S21. The equivalent circuit fit model for figures 5 and S22 Nyquist data.

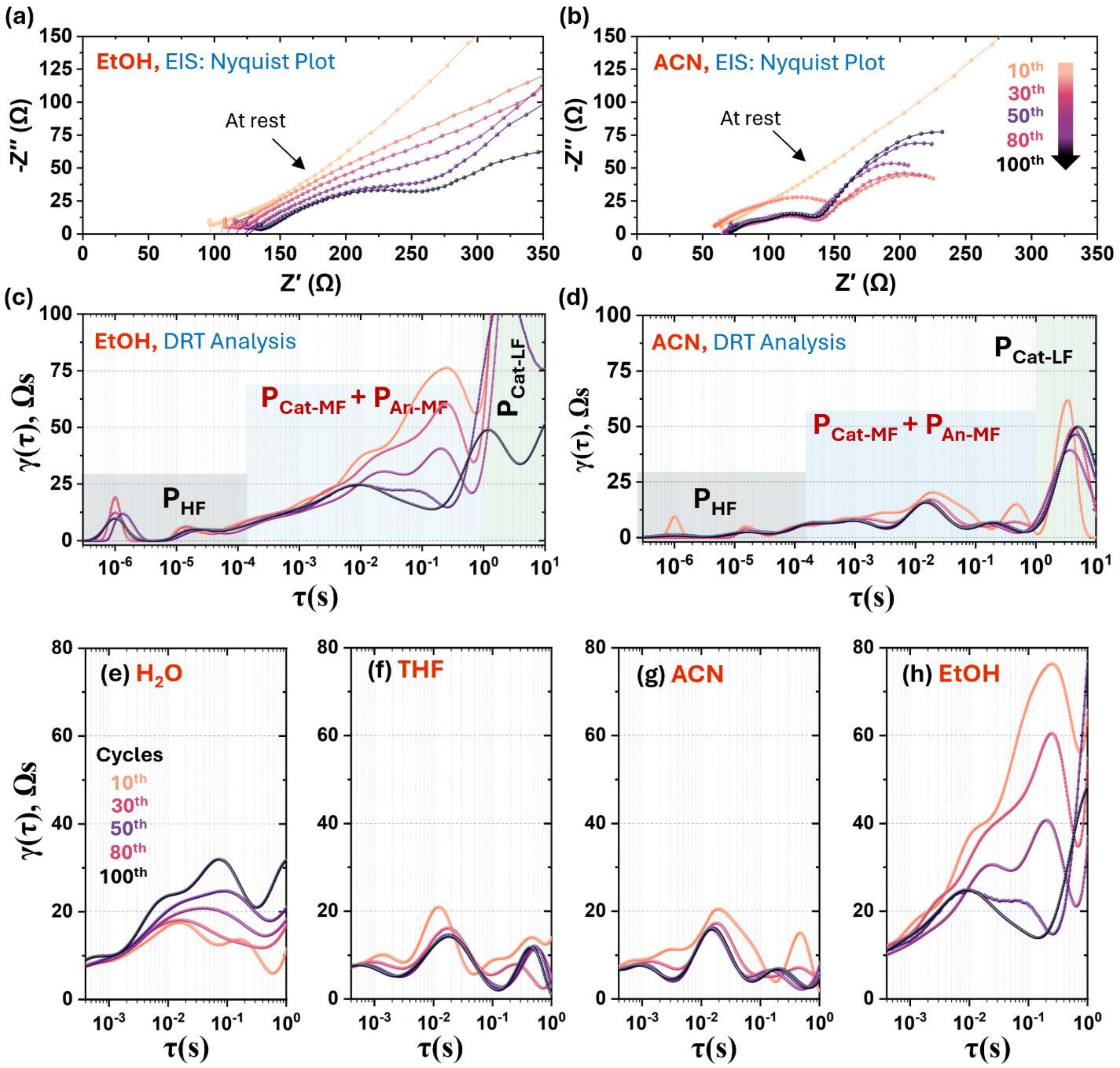

Figure S22. Nyquist impedance evolution of Li-In|Li_6PS_5Cl||Li_3InCl_6|NMC622 all-solid-state cells during cycling at 0.2C with the Li_3InCl_6 SE synthesized from (a) EtOH, (b) ACN. (c, d) Corresponding DRT analysis. The 2D DRT convolutions corresponding to the $P_{Cat\text{-}MF}$ region over the long-term cycling for Li_3InCl_6 SE synthesized from (e) H_2O, (F) THF (g) ACN and (h) EtOH

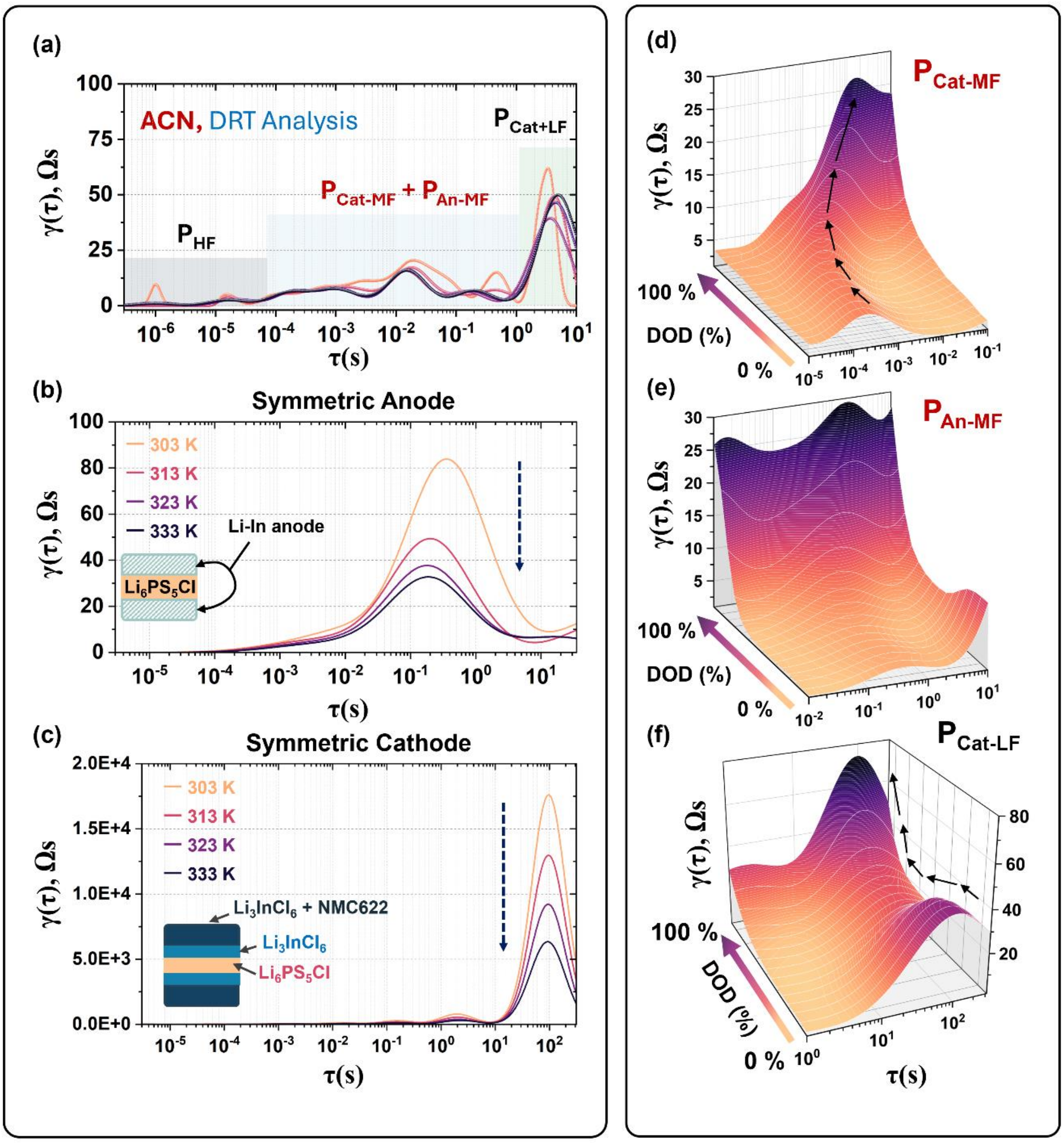

Figure S23. (a) DRT evolution Li-In|Li_6PS_5Cl||Li_3InCl_6|NMC622 during cycling at 0.2C with the Li_3InCl_6 SE synthesized from ACN. Temperature dependence of DRT evolution for ASSBs in symmetric configuration, e.g., (b) Li-In|Li_6PS_5Cl|Li-In cell and (c) NMC622| Li_3InCl_6||Li_6PS_5Cl|Li_3InCl_6|NMC622. The DOD/SOC dependence of the 2D DRT convolutions corresponding (d) $P_{Cat\text{-}MF}$, (e) $P_{An\text{-}MF}$, and (f) $P_{Cat\text{-}LF}$ regions for a representative Li-In|Li_6PS_5Cl ||Li_3InCl_6|NMC622 cell during discharge at 0.2 C current equivalent.

Assignment of components in DRT analysis:

We conducted a series of experiments to verify the DRT components assignment (**Figure S23a**; a representative full-cell DRT profile), involving the following scenarios. Two symmetric all-solid-state battery (ASSB) cells were prepared with the following configurations - Li-In|Li_6PS_5Cl|Li-In and NMC622+LIC| LIC|Li_6PS_5Cl|LIC|NMC622+LIC, where LIC stands for

Li_3InCl_6. The impedance response was measured in the 1 MHz to 10 μHz frequency range at varied temperatures ranging from 30°C (303 K) to 60°C (333 K), and the corresponding DRT profile is shown in **S23b** and **S23c**, respectively.

In accordance with the work on DRT deconvolution of impedance spectra for solid-state batteries with Li_6PS_5Br[5], our Li_6PS_5Cl-based symmetric cell shows the temperature dependence of its major component in the 0.1 (10^{-1}) to 1 s τ range (**Figure S23b**). Due to the stable Li-In - Li_6PS_5Cl interface and the absence of any significant DRT component in the low to lower mid-frequency region for the symmetric anode configuration, which was also noted by Hori *et al*. for Li_6PS_5Br, we conclude that the anode-electrolyte interfacial impedance is significantly low and does not prominently appear in the full-cell DRT.

Similarly, in the cathodic symmetric configuration (**Figure S23c**), the variation observed in the DRT plot is confined to the high τ or low-frequency region, which has been assigned to signify the changes associated with the cathodic charge transfer component.

Further, the DRT deconvolution over varied SOC/DOD in the case of a representative THF-based cell after a significant number of cycles (**Figures S23d-f**) highlights that the majority of resistive interaction in the mid (10^{-4} to 1 s in τ scale) to low-frequency (> 1 s in τ scale) regions manifests from the overall cathode impedance and the impedance associated with the cathode interfacial charge transfer, respectively.

It is important to note that DRT, being a mathematical tool for reverse Fourier analysis of the impedance response, can overestimate or underestimate the number of deconvolution characteristic components corresponding to the electrochemical interaction components in an actual physical system.

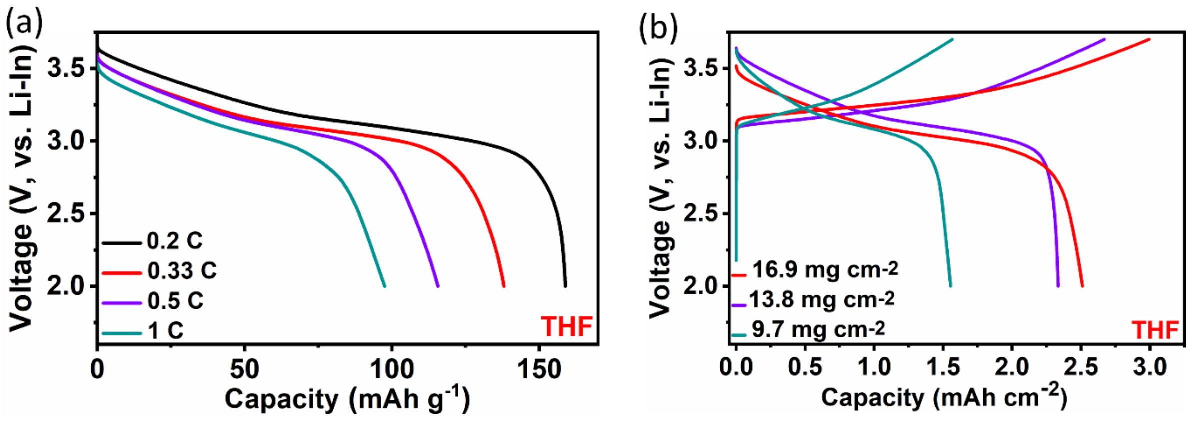

Figure S24. (a) RT discharge rate capability of Li-In|Li_6PS_5Cl||Li_3InCl_6|NMC622 cell at 0.2C,0.33C, 0.5C, and 1C for pristine Li_3InCl_6 SSE synthesized in THF. (b) Charge/discharge curves of high-loading cells with Li_3InCl_6 SE synthesized using THF.

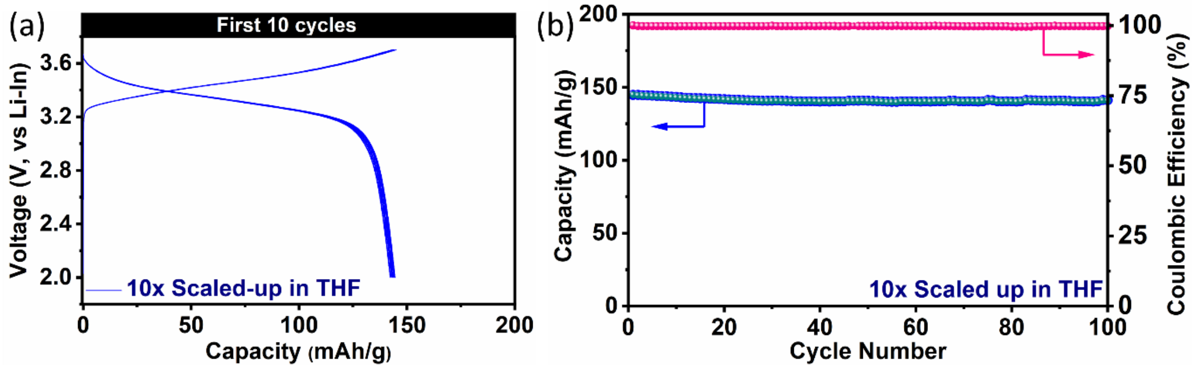

Figure S25. (a) First ten charge–discharge curves for Li-In|Li_6PS_5Cl||Li_3InCl_6|NMC622 cell in RT at 0.2C for 10x Scaled up Li_3InCl_6 SE synthesized using THF. (b) RT cycling stability of Li-In/Lii_6PS_5CL/Li_3InCl_6/NMC622 at 0.2C based on 10x Scaled up Li_3InCl_6 SE synthesized using THF.

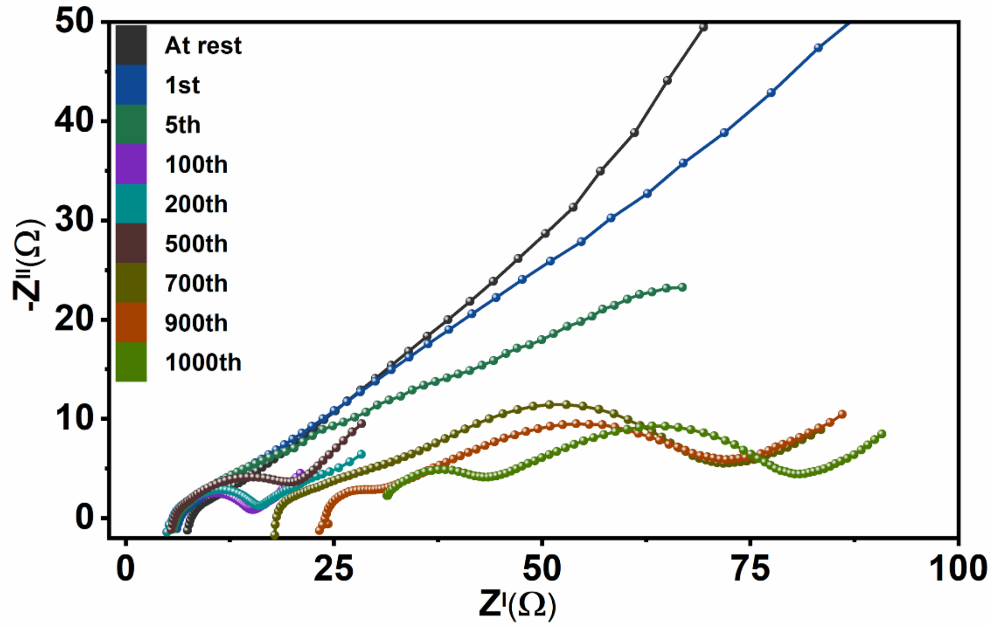

Figure S26. Nyquist impedance evolution of the Li-In|Li_6PS_5Cl||Li_3InCl_6|NMC622 cell with Li_3InCl_6 SE synthesized using THF at 60°C over long-term cycling at 2C.

References

[1] C. Hänsel, P. V. Kumar, D. Kundu, *Chem. Mater.* **2020**, *32*, 10501–10510.

[2] C. Hänsel, D. Kundu, C. Hänsel, D. Kundu, *Adv. Mater. Interfaces* **2021**, *8*, 2100206.

[3] A. Maradesa, B. Py, T. H. Wan, M. B. Effat, F. Ciucci, *J. Electrochem Soc.* **2023**, *170*, 030502.

[4] T. H. Wan, M. Saccoccio, C. Chen, F. Ciucci, *Electrochim. Acta* **2015**, *184*, 483–499.

[5] S. Hori, R. Kanno, X. Sun, S. Song, M. Hirayama, B. Hauck, M. Dippon, S. Dierickx, E. Ivers-Tiffée, *J Power Sources* **2023**, *556*, 232450.

[6] B. Kisan, R. K. Bhuyan, R. K. Mohapatra, *Nano-biosorbents for Decontamination of Water, Air, and Soil Pollution* **2022**, 529–550.

[7] V. S. Vinila, J. Isac, *Design, Fabrication, and Characterization of Multifunctional Nanomaterials* **2021**, 319–341.

[8] X. Li, J. Liang, J. Luo, M. Norouzi Banis, C. Wang, W. Li, S. Deng, C. Yu, F. Zhao, Y. Hu, T. K. Sham, L. Zhang, S. Zhao, S. Lu, H. Huang, R. Li, K. R. Adair, X. Sun, *Energy Environ. Sci.* **2019**, *12*, 2665–2671.

[9] K. Wang, Q. Ye, J. Zhang, H. Huang, Y. Gan, X. He, W. Zhang, *Front Mater.* **2021**, *8*, 727617.

[10] P. Molaiyan, S. E. Mailhiot, K. Voges, A. M. Kantola, T. Hu, P. Michalowski, A. Kwade, V. V. Telkki, U. Lassi, *Mater. Des.* **2023**, *227*, 111690.

[11] T. Ma, Z. Wang, D. Wu, P. Lu, X. Zhu, M. Yang, J. Peng, L. Chen, H. Li, F. Wu, *Energy Environ. Sci.* **2023**, *16*, 2142–2152.

[12] S. Wen, H. Sheng, Y. Zhang, S. Zheng, Z. Wang, *J. Alloys Compd.* **2024**, *984*, 173973.

[13] X. Li, J. Liang, N. Chen, J. Luo, K. R. Adair, C. Wang, M. N. Banis, T. K. Sham, L. Zhang, S. Zhao, S. Lu, H. Huang, R. Li, X. Sun, *Angew. Chem. Int. Ed.* **2019**, *58*, 16427–16432.

[14] X. Luo, D. Cai, X. Wang, X. Xia, C. Gu, J. Tu, *ACS Appl Mater. Interfaces* **2022**, *14*, 29844–29855.
